# Analysis of Physicochemical and Structural Properties Determining HIV-1 Coreceptor Usage

**DOI:** 10.1371/journal.pcbi.1002977

**Published:** 2013-03-21

**Authors:** Katarzyna Bozek, Thomas Lengauer, Saleta Sierra, Rolf Kaiser, Francisco S. Domingues

**Affiliations:** 1 Max Planck Institute for Computer Science, Saarbrucken, Germany; 2 Institute of Virology, University of Cologne, Cologne, Germany; 3 Center for Biomedicine, EURAC, Bolzano, Italy; University of California San Diego, United States of America

## Abstract

The relationship of HIV tropism with disease progression and the recent development of CCR5-blocking drugs underscore the importance of monitoring virus coreceptor usage. As an alternative to costly phenotypic assays, computational methods aim at predicting virus tropism based on the sequence and structure of the V3 loop of the virus gp120 protein. Here we present a numerical descriptor of the V3 loop encoding its physicochemical and structural properties. The descriptor allows for structure-based prediction of HIV tropism and identification of properties of the V3 loop that are crucial for coreceptor usage. Use of the proposed descriptor for prediction results in a statistically significant improvement over the prediction based solely on V3 sequence with 3 percentage points improvement in AUC and 7 percentage points in sensitivity at the specificity of the 11/25 rule (95%). We additionally assessed the predictive power of the new method on clinically derived ‘bulk’ sequence data and obtained a statistically significant improvement in AUC of 3 percentage points over sequence-based prediction. Furthermore, we demonstrated the capacity of our method to predict therapy outcome by applying it to 53 samples from patients undergoing Maraviroc therapy. The analysis of structural features of the loop informative of tropism indicates the importance of two loop regions and their physicochemical properties. The regions are located on opposite strands of the loop stem and the respective features are predominantly charge-, hydrophobicity- and structure-related. These regions are in close proximity in the bound conformation of the loop potentially forming a site determinant for the coreceptor binding. The method is available via server under http://structure.bioinf.mpi-inf.mpg.de/.

## Author Summary

Human Immunodeficiency Virus (HIV) requires one of the chemokine coreceptors CCR5 or CXCR4 for entry into the host cell. The capacity of the virus to use one or both of these coreceptors is termed tropism. Monitoring HIV tropism is of high importance due to the relationship of the emergence of CXCR4-tropic virus with the progression of immunodeficiency and for patient treatment with the recently developed CCR5 antagonists. Computational methods for predicting HIV tropism are based on sequence and on structure of the third variable region (V3 loop) of the viral gp120 protein — the major determinant of the HIV tropism. Limitations of the existing methods include the limited insights they provide into the biochemical determinants of coreceptor usage, high computational load of the structure-based methods and low prediction accuracy on clinically derived patient samples. Here we propose a numerical descriptor of the V3 loop encoding the physicochemical and structural properties of the loop. The new descriptor allows for server-based prediction of viral tropism with accuracy comparable to that of established sequence-based methods both on clonal and clinically derived patient data as well as for the interpretation of the properties of the loop relevant for tropism. The server is available under http://structure.bioinf.mpi-inf.mpg.de/.

## Introduction

The entry of the human immunodeficiency virus (HIV) into human cells is initiated by binding of the viral envelope glycoprotein gp120 to the cellular CD4 receptor [1], [2]. This primary interaction induces conformational changes in gp120 [3] that enable viral binding to one of the cell-surface coreceptors CCR5 or CXCR4 [4]. The interaction of gp120 with the coreceptor induces a series of further rearrangements in the envelope glycoproteins that trigger fusion of the virus and cell membranes [1]. The third variable region (V3) of gp120 [5], [6] plays a crucial role in biding to the coreceptor. Whether a virus can bind to CCR5 only (R5 virus), or is capable of binding to CXCR4 (X4 virus) is determined predominantly by the sequence and structure of this region [7]. The phenotype of viral coreceptor usage is termed viral tropism.

It has been shown that in the early, asymptomatic stages of infection mainly R5 viruses are observed, whereas progression towards AIDS is often associated with the emergence of X4 viruses [8]. The finding that humans who lack CCR5 expression due to the homozygosity of the Δ32 mutation in the CCR5 gene are resistant to HIV-1 infection [9] stimulated research on CCR5 inhibitors which led to the licensing of Maraviroc (MVC) [10] for clinical use in 2007. Viral tropism is an indicator of disease progression and determining viral tropism is a companion diagnostic obligatory for the application of CCR5 inhibitors. Therefore there is a need for efficient methods for monitoring of coreceptor usage and for a better understanding of its determinants.

Computational methods for predicting viral tropism based on the sequence of the V3 loop have been developed [11], [12], [13], [14] as an alternative to costly phenotypic assays for testing of the coreceptor usage [15]. The 11/25 rule was proposed as an initial approach for inferring coreceptor usage, and is based on the observation that a positive charge on either of the 11^th^ or 25^th^ residues in the V3 region is indicative of an X4 virus [5], [6]. Due to its simplicity, the 11/25 rule has been commonly used although it has been shown that for many viral variants, changes at positions 11 or 25 are neither necessary nor sufficient for the tropism switch [11]. More elaborate sequence-based methods for prediction of coreceptor usage rely on a binary encoding of amino acids in the V3 sequence and use statistical approaches to construct predictive models and to infer residues strongly related to the tropism [11], [12], [13], [14]. The geno2pheno[coreceptor] method developed by our lab has been made freely available on the internet and is widely used throughout Europe and beyond for interpreting genotypic data measured as a companion diagnostic to Maraviroc therapy. The method has entered the German/Austrian expert guidelines for HIV-1 tropism testing in 2009 [16] and the respective European guidelines in 2011 [17]. The major drawback of the binary sequence representation is that it only indirectly encodes the physicochemical properties of amino acids and their spatial arrangement in the binding site which ultimately determine viral tropism.

Structures of gp120 including the V3 loop have been determined by x-ray crystallography [18], [19]. The V3 loop is an extended structure protruding approximately 30 Å from the CD4-bound core of gp120 [18]. It is composed of a conserved base, a flexible stem that rigidifies upon coreceptor binding and a tip in a β-hairpin conformation. After the first structure of the V3 loop has been resolved [18], new coreceptor prediction methods were developed [20], [21] incorporating structural information on the loop in the prediction process. Sander et al. [21] proposed a distance-based descriptor of the spatial arrangement of physicochemical properties of the loop. They found that the distance information resulting from structural modeling of the side chains of the loop together with a binary encoding of its sequence outperforms prediction methods based on sequence alone. Dybowski et al. [20] developed a two-level classification approach that combines two physicochemical properties of the loop – electrostatic potential and hydrophobicity. This two-level approach resulted in improvement in prediction accuracy over prediction based on sequence alone. Even though including the structural information into the prediction represents a step forward in understanding of the binding mechanism of gp120 to the coreceptor, both methods have limitations. The method by Sander et al. is based on molecular distances that do not offer a direct interpretation of the structural determinants of the phenotype. Dybowski et al. include only two features in their predictor while a systematic analysis of a larger set of physicochemical features of the V3 loop would allow for identifying other features relevant for tropism. Both methods involve costly computational operations such as calculation of the electrostatic potential or modeling of side chains that stand in the way of making the methods available as an online application. Finally, all previously proposed methods except one [14] were developed and tested exclusively on clonal data. Such data are inferred from lab-cloned viruses as opposed to clinically derived data, which are obtained through bulk Sanger sequencing of patient samples and contain viral mixtures. In bulk sequencing data, diversity of virus populations in a patient is represented by a consensus sequence comprising dominant strains. The exact composition of the virus population as well as viral minorities below 10% [22] of the population are not detected by bulk Sanger sequencing which has been shown to pose additional challenge for *in silico* coreceptor prediction [14].

The work presented here was motivated by the goal of developing a method for genotypic prediction of viral tropism that is at least as accurate as existing structure-based methods, i.e., more accurate than the widely used sequence-based method [14]. At the same time, the method should allow for a computationally efficient implementation allowing for its general use as an online application.

To meet this goal we present a systematic approach to incorporating physicochemical and structural properties of the V3 loop into the prediction of HIV coreceptor usage. We map 54 amino acid indices representing the physicochemical properties of amino acids onto the V3 loop structure and use methods from statistical learning to extract those features that are most informative of coreceptor usage. The extracted set of features represents a small fraction of the initial feature set and models based on this set attain higher prediction accuracy with decreased computational load. Our structural descriptor affords direct interpretation of the features of the V3 loop relevant for viral tropism by pointing to specific physicochemical properties of amino acids in different parts of the loop being predictive of coreceptor usage. We also applied our method to clinically derived (bulk) data and tested its usability for prediction of the MVC therapy outcome.

## Results

### Model parameters

**Figure 1:**
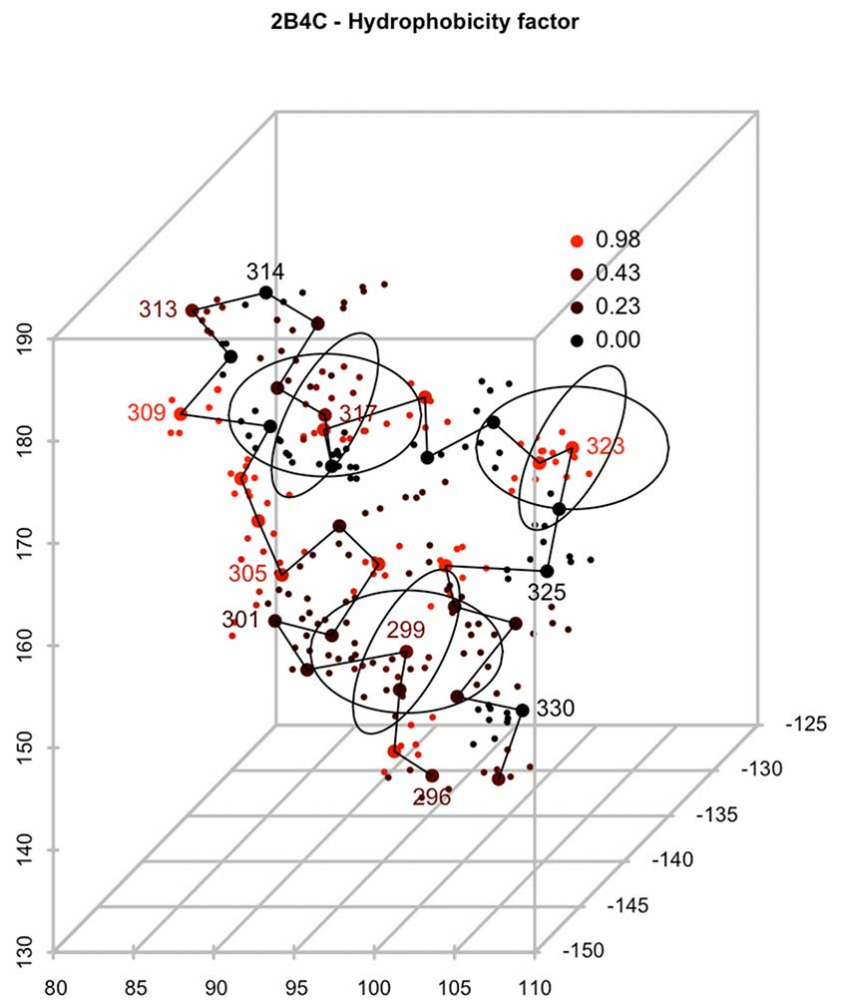
Schematic illustration of the sphere-shaped proximities of the structural descriptor. Atoms of the 2B4C V3 loop structure are represented by dots, representative atoms by larger dots. The black line connects representative atoms of the adjacent loop residues. Atoms of each residue are colored according to the “Hydrophobicity factor” amino acid index. Spheres are centered at the representative atoms of the loop residues. The spheres for residues 299, 317 and 323 are shown. The physicochemical features of residues within each sphere were averaged and used as a part of the structural descriptor.

The structural descriptor of the V3 loop is based on the published structure of the V3 loop [18] and amino acid indices [23] representing physicochemical properties of amino acids in a numerical form. Each residue of the V3 loop sequence is represented by a vector comprising the 56 preselected indices [24]. The residue positions were mapped to spheres centered along the V3 loop backbone (Figure 1). The spheres represent structural proximities along the loop as well as uncertainty in the structural conformation of individual loop variants. The vectors of amino acid indices of the mapped residues were normalized using Gaussian smoothing and summed up within each sphere. Next, the sphere vectors were concatenated into a single V3 loop vector. This vector was used as the V3 loop structural descriptor in the statistical model (*full model*) for coreceptor usage prediction. A training dataset of 1186 phenotyped V3 sequences from the Los Alamos database [25] (*clonal dataset*) was used for model development.

We investigated the average number of residues covered by each sphere and selected the radius of 8 Å based on predefined criteria (see Text S1, Figure S1). We tested several other radii for their predictive performance (Figure S2). Throughout this study we used area-under-receiver-operating-characteristic (ROC) curve (AUC) and, in line with the common approach to validating genotypic predictions of viral tropism, sensitivity at the specificity of 11/25 rule in a given dataset (ranging between 0.89 and 0.97 termed here *sensitivity* for brevity) calculated in a 10×10-fold cross validation as cutoff-independent measures of the prediction accuracy. The radius of 8 Å yielded the AUC of 0.847 and a sensitivity of 0.587. Smaller or larger radii led to significant reduction of prediction performance (p<0.001 for R = 3 Å and R = 15 Å, paired Wilcoxon test). The performances of models with different parameter values are shown in Figure S2.

For comparison we implemented two sequence-based descriptors of the V3 loop. The *g2p model* represents each amino acid as a binary vector of size 20 in which the position of a single 1 indicates the amino acid it encodes. This representation is used by sequence-based approaches, among others by geno2pheno[coreceptor] [14]. Those approaches are not necessarily based on the same training sets as the one used in our study. Certainly the training set used by the geno2pheno[coreceptor] method is different. The *aaindex model* encodes each amino acid as a vector of the 56 amino acid indices used in the structural descriptor.

### Feature selection

In order to reduce the highly redundant feature vector of the structural descriptor of the full model and to investigate which features are informative for coreceptor usage we applied several feature selection procedures: Random Forest (RF) [26], linear support vector machine (SVM) [27] and Lasso regression [28]. We next compared the performance of the full model based on the entire set of features to the descriptor based on separate and combined subsets of features selected by three feature selection methods. SVM was the prediction method used throughout this study independent of the feature set and sequence encoding. Overall, reducing the feature set resulted in improved prediction accuracy over the full set of features and over the g2p model (Table 1). The SVM(1) model based on the top 1% ranking features performed better than the SVM(5) model based on a larger feature set of the top 5% ranked features. The Lasso model based on the most strongly reduced feature set (102 features) selected via Lasso regression resulted in the highest performance with AUC 0.893 and sensitivity 0.674. Models based on features selected via RF ranking showed the poorest predictive performance of all models tested (Table 1).

**Table 1:** Performance of models based on feature sets and combination of feature sets selected using different feature selection methods.*

model	features	AUC	sensitivity
g2p	1000	0.860	0.616
aaindex	2800	0.829	0.565
full	5544	0.847	0.587
RF(1)	144	0.860	0.673
RF(5)	241	0.863	0.634
**SVM(1)**	**123**	**0.889**	**0.706**
SVM(5)	362	0.879	0.674
**Lasso**	**102**	**0.893**	**0.674**
RF(1)_SVM(1)	264	0.878	0.683
RF(5)_SVM(5)	588	0.875	0.668
RF(1)_Lasso	226	0.883	0.652
RF(5)_Lasso	315	0.874	0.639
**SVM(1)_Lasso^#^**	**218**	**0.892**	**0.686**
SVM(5)_Lasso	448	0.881	0.674
RF(1)_SVM(1)_Lasso	340	0.868	0.644
RF(5)_SVM(5)_Lasso	653	0.883	0.685

* Models are named after the feature selection method with the number in parentheses indicating the percentage cutoff of the ranked features. Sensitivity shown is at specificity of 11/25 rule in the clonal dataset. The clonal model is indicated with a #.

The sets of features selected by the three feature selection methods show a limited overlap. The initial feature set contains small subsets of highly correlated features that pertain to highly correlated amino acid properties in overlapping structure regions (spheres). These features convey the same information to the prediction method and can be therefore selected interchangeably by each method (see Text S2 and Figure S3). However, the overall correlation of features in the descriptor is low and a version of the descriptor based on an uncorrelated feature set does not yield improved performance (Text S3, Figure S4). We performed the same feature selection procedures on models based on the structural descriptor with a sphere radius of 10 Å, chosen based on analysis described in Text S1 and Figure S1. The results of models based on this radius showed similar patterns of performance although with lower prediction performance (data not shown). In the rest of this study we used models based on the 8 Å radius.

### Clonal model

**Figure 2:**
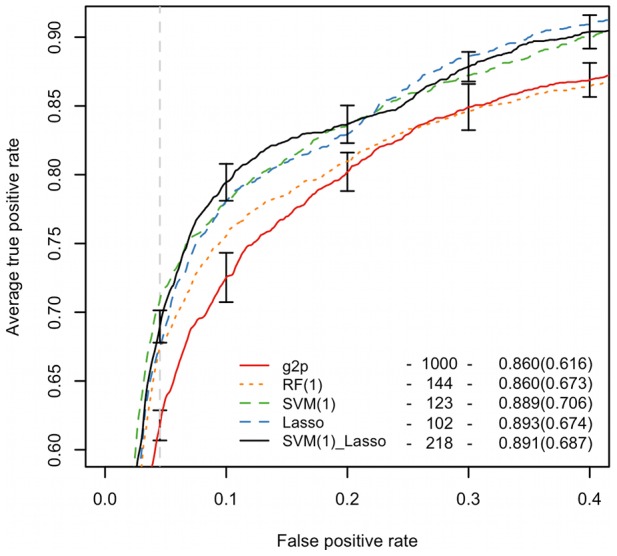
Performance of models based on features selected using RF, SVM and Lasso. ROCR of different models are plotted. The legend lists the number of features, AUC and sensitivity at the specificity of the 11/25 rule (6.28%. indicated by the vertical dashed line) in brackets. The clonal model is represented by a black solid line. Vertical segments show the standard deviations of the 10×10-fold cross validation curves of the clonal and g2p models. Comparison of clonal and g2p models via precision-recall curves is shown in the Figure S14.

We inspected the predictive performance of models based on combined sets of features selected using different methods (Table 1). Given the performance of the tested models we selected the SVM(1)_Lasso model based on the combined set of the top 1% SVM-ranked features and the Lasso-selected features, termed it *clonal model* and used as the structural descriptor model in subsequent tests (Figure 2). The performance of the clonal model was not significantly higher than the performance of the Lasso model, however we chose the SVM(1)_Lasso feature set offering higher sensitivity. The AUC and sensitivity of the clonal model were significantly higher than those of the sequence-based g2p and aaindex models (p<0.01, paired Wilcoxon test). In our approach the features were first selected and then evaluated on the entire sequence set in two subsequent steps involving cross validation. A test involving reselection of the SVM(1)_Lasso features in each cross validation run (nested cross validation) resulted in a decrease of the AUC of only approximately 1.6 percentage points. Since the analysis of features selected for the clonal model was an additional goal of this study, we refrained from reselection of features within the separate cross validation runs. We regard this difference in performance as a potential uncertainty of our accuracy estimation inherent to the feature selection procedure. The accuracy obtained using nested cross validation is still significantly higher (p∼0.003) than the accuracy of the g2p prediction suggesting that the selected structural and physicochemical features are more informative of tropism than the sequence alone [14].

In our approach we refrain from modelling of the side chains of the V3 loop. There is certain level of imprecision related to modelling of side chains due to the high flexibility and variability of the loop. We use our approach based on spheres as an approximation of the real structure of the loop that is costly to derive computationally and is unreliable. Such approximation of the structure is robust against indels as we observe no relationship of the model performance to the presence of indels in a sequence (Figure S5).

We additionally tested the performance of a model based on a different V3 loop structure (Protein Data Bank (PDB) code 2QAD) [19] and performance of models based on combinations of structure- and sequence-based descriptors. However, these models did not yield an improvement in prediction performance (see Text S4).

### Comparison with other structure-based methods

We compared the performance of our method with the performance of previously published structure-based methods for tropism prediction [20], [21] by testing the descriptor on the datasets used in the study of Sander et al. [21] (*Sander dataset*) and the study of Dybowski et al. [20] (*Dybowski dataset*). These datasets have sequence overlap of 19% and 58% with the clonal dataset, respectively. The overlap varies presumably due to different content of the Los Alamos database [25] at a given time point and to the different filtering methods used. In order to avoid overtraining and to test the performance of our model independently of the training dataset, we did not repeat feature selection on the datasets of other studies but based the predictions on the features of the clonal model. Structural descriptors trained on the Sander and Dybowski datasets were constructed based on these features and tested in a 10×10-fold cross validation setting. The clonal model showed performance similar to that of the original method of Sander et al. with a lower AUC 0.901 (0.923 reported by Sander et al.) and higher sensitivity 0.782 (0.774 reported by Sander et al.), see Table 2. The result of Sander et al. was obtained on a dataset with no insertions or deletions relative to the reference structure and involved costly side-chain modeling steps. In contrast, our result was based on features selected on a different dataset, and the prediction procedure did not involve structural modeling. The clonal model reached better performance on the Dybowski dataset in comparison to the original method with AUC 0.948 (0.937 reported by Dybowski et al.) and sensitivity 0.838 (0.810 reported by Dybowski et al.), see Table 2.

**Table 2:** Performance of the clonal and clinical models on different datasets.*

dataset	model	features	AUC	sensitivity	original method/g2p	
					AUC	sensitivity
Sander	clonal	218	0.901	0.782	0.923	0.774
Dybowski	clonal	218	0.948	0.838	0.937	0.810
HOMER	clinical	59	0.774	0.463	0.743	0.451
HOMER-clinical	clinical (CD4, VL)	61	0.803	0.474	0.781	0.442

* The performance of the clonal model on the Sander and Dybowski datasets is compared to the performance of the original methods [20], [21] developed on these datasets. The clinical model constructed on the HOMER dataset is compared to the performance of g2p method in a 10×10-fold cross validation. Additionally the clinical model trained on the HOMER dataset coupled with clinical correlates (VL and CD4^+^ T cell counts) (HOMER-clinical) is compared to the g2p model also coupled with clinical correlates.

### Performance on clinical data

**Figure 3:**
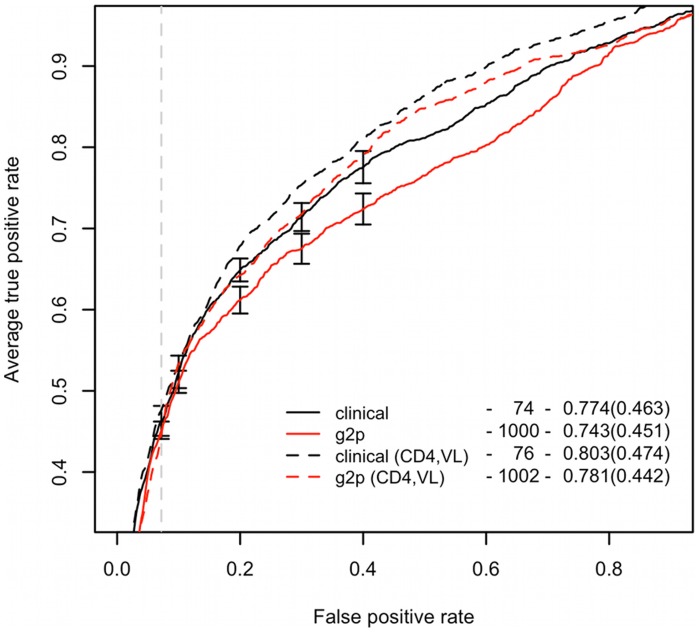
Performance of the clinical model compared to the g2p model. Black curves represent the clinical model, red – the g2p method. Dashed curves represent models enhanced with clinical patient information. The vertical dashed line indicates the specificity of the 11/25 on the clinical dataset. Vertical segments show the standard deviations of the 10×10-fold cross validation curves of the clinical and g2p models. The legend presents the results as in Figure 2.

We additionally tested the method on clinically derived patient data from the HOMER cohort [29] (*HOMER dataset*). We reran the feature selection procedures on this dataset and selected the best performing model (Lasso) as the *clinical model*. The clinical model showed AUC 0.774 and the sensitivity 0.463 [29], a result significantly higher (p<0.01, paired Wilcoxon test) than the one of the g2p model [14] with AUC 0.743 and sensitivity of 0.451 (Table 2, Figure 3). To support this assessment, we performed an additional test on another independent patien-derive sequence set. On this dataset we observed a similar performance advantage of the clinical over the g2p model (see Text S5, Figure S6). Similar to the clonal model, the test of reselecting the Lasso features within the cross validation runs resulted in a decrease of performance of approximately 1.8% in AUC, which is still significantly higher (p∼0.002) than the performance of the g2p model.

We additionally tested the effect of amino-acid ambiguities on the prediction accuracy of the clinical model and found that the combined information from both types of sequence positions, ambiguous and non-ambiguous is important for tropism prediction (see Text S6 and Table S1).

As shown by Sing et al. [14] accuracy of tropism prediction methods applied on clinical data improves upon augmenting the sequence information with clinical correlates, such as VL or CD4^+^ T cell counts. Accordingly, adding such clinical information as additional features to the input of the clinical model significantly improved the predictive performance (p<0.001, paired Wilcoxon test) over that of the clinical model to AUC 0.803 and sensitivity 0.474 (Table 2, Figure 3). This performance is also significantly higher than that of the g2p model containing clinical correlates in terms of both AUC and sensitivity (p<0.001, paired Wilcoxon test). This demonstrates the higher prediction accuracy of the new method based on preselected structural and physicochemical features of the V3 loop over the commonly used sequence-based methods such as geno2pheno [14].

### Predicting therapy outcome

Finally, we tested the prediction performance of the clinical model on a dataset of sequences collected at therapy start from a German cohort of patients undergoing MVC therapy (*MVC dataset*). Among the 53 sequences five originate from patients who experienced therapy failure. With the decision cutoff at the 11/25 rule specificity of the HOMER dataset (specificity 0.928, score 0.097) three of these sequences were predicted as X4 viruses in accordance with the patient therapy outcome. The two remaining sequences of patients experiencing therapy failure that were predicted as R5 viruses were also phenotyped as R5 virus, which suggests the presence of undetectable minorities as the potential reason for the classification error.

The remaining 48 patients experienced therapy success. 41 of the cases were classified as R5 viruses by the clinical model, which is in accordance with the patient therapy outcome. Out of seven remaining cases that were classified as X4 viruses, two were phenotyped as X4 viruses. For comparison, we predicted tropism of the sequences in this dataset using the g2p model. This sequence-based prediction reported correctly only two therapy failure cases but also 44 therapy success cases which is more than the clinical model predicted. As a measure of the quality of predictions of the MVC dataset we used the Matthews correlation coefficient (MCC), which quantifies the correlation of the observed and predicted binary classification and is suited for datasets with an unbalanced class proportion. Therapy outcome prediction based on structural descriptor showed overall accuracy of MCC = 0.34 comparing favorably with g2p model yielding MCC = 0.29.

Phenotypic characterization was only available for a subset of 28 sequences from the MVC dataset (Trofile dataset). In this subset the phenotype appeared to be the best predictor of the therapy outcome with one correctly predicted therapy failure case out of three and 23 correctly predicted therapy successes out of 25 (MCC = 0.25). The clinical model reported the same number of correctly predicted therapy failure cases and lower number of 20 correctly predicted therapy success cases (MCC = 0.10). The clinical model scored higher than the g2p method that did not report correctly any of the therapy failure cases and predicted correctly 23 therapy successes (MCC = −0.10). Additionally, the clinical model correctly classified all X4 sequences in the Trofile dataset reaching MCC of 0.660 and favorably comparing with the g2p showing MCC of 0.352.

Overall, the phenotype as well as the structural descriptor model and the g2p model trained on clonal data showed a generally lower capacity of detecting therapy outcome compared to the models trained on clinical data. Detailed results of the MVC dataset analysis are provided in Tables S2 and S3.

### Feature clustering

**Figure 4:**
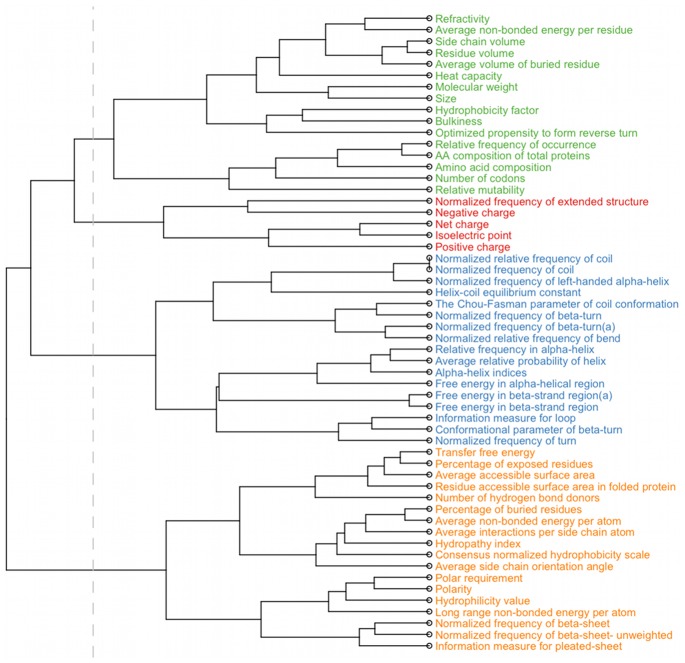
Hierarchical clustering of the amino acid indices. The vertical line indicates the separation of the tree into four clusters analyzed in this study. Labels of the tree are colored according to the clusters.

In order to facilitate the interpretation of the large number of selected features we clustered the 56 amino acid indices into four groups (Figure 4) using hierarchical clustering. Cluster 1 is composed of two types of indices – related to residue size and volume and to residue occurrence in proteins. Cluster 2 contains the smallest number of indices and is composed of indices related to residue charge. Indices of cluster 3 are related to the secondary and tertiary structure of proteins. Cluster 4 contains indices related to different structural properties e.g. residue occurrence in β-sheet, solvent accessibility, amino acid polarity or hydrophobicity.

### Feature analysis

By combining amino-acid indices with specific positions on the V3 loop, the proposed features can be interpreted in terms of physicochemical properties along the structure of the loop. The features selected for the clonal model are informative about the coreceptor usage. Their analysis can therefore provide insights into the physicochemical and structural factors of viral tropism.

#### Features selected by different methods

Features of the clonal model were selected based on two different feature selection methods – Lasso and SVM. Among 218 features in this model seven were selected by both methods. Three of the features describe electrical charge at positions 319–322. Two of the features are structure-related (“Free energy in β-strand region”, “Normalized frequency of turn”) and related to positions 304 and 305. These amino acid indices based on statistical analysis of 3D structures define propensities of amino acids to form β-strands [30] and reverse turns [31], respectively. The remaining two features selected by both methods are based on amino-acid indices “Number of codons” at position 297 and “Relative mutability” at position 307. “Number of codons” defines how many different codons encode a given amino acid and “Relative mutability” quantifies the rate of exchange of an amino acid based on alignment of a large set of protein sequences [32].

#### Top-ranking features

**Figure 5:**
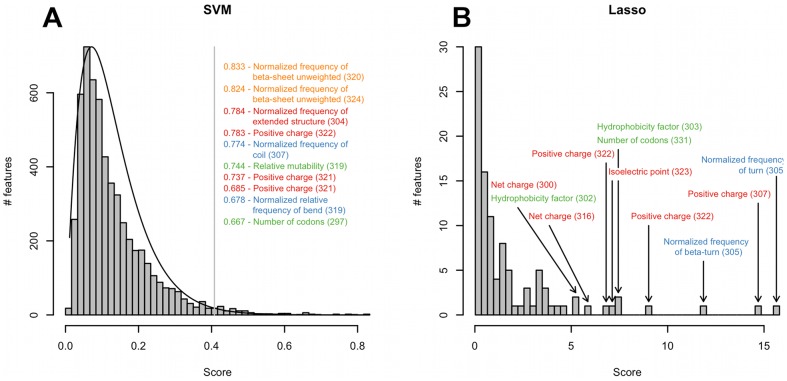
Distribution of scores of features selected using SVM and Lasso. (A) Distribution of scores of the SVM method. The vertical line indicates the cutoff for the selection of features for the clonal model. The scores of the top-scoring features are listed. (B) Distribution of scores of the Lasso method. Top-scoring features in the distribution are indicated. On both panels, positions of the features mapped on the V3 loop structure are indicated in brackets, labels are colored according to the clusters shown in Figure 4.

Both feature selection methods allow for feature ranking based on the feature coefficients in the respective linear models. We inspected the top-scoring features in both rankings (Figure 5). SVM scoring follows a gamma distribution with a shape parameter of 2.2. The selected features follow a close to uniform distribution in the range of values above the chosen cutoff. The feature scores based on Lasso selection are distributed over a wider range of values and contain several high-scoring outliers.

Among the top-scoring features selected by both methods we found “Positive charge” at the stem position 322 corresponding to the position 25 in the consensus sequence. Highly ranked features in the SVM scoring include also “Positive charge” at the position 321. Additionally SVM scoring pointed to secondary structure propensities and mutability at the loop stem (“Normalized frequency of coil”, “Normalized frequency of β-sheet unweighted”, “Normalized relative frequency of bend” at positions 307, 319–320 and 324). These indices are based on statistical analyses of secondary structures and statistical models for predicting tertiary structures and define the contributions of different amino acids to the formation of a given structural element [33], [34], [35].

Among the high-ranking features in the Lasso scoring we found predominantly charge indices at the loop stem (“Positive charge”, “Isoelectric point” and “Net charge” at positions 307, 316, 322–323) and at the loop base (“Net charge” at position 300). Additionally we found “Hydrophobicity factor” at the loop base positions 302–303. Two structure-related features based on “Normalized frequency of turn” and “Normalized frequency of β-turn” amino acid indices at the base position 305 were also scored high by the Lasso method. Details of the feature ranking are provided as Tables S4, S5 and S6.

#### Amino-acid indices and their clusters

Next, we investigated which clusters of indices were significantly overrepresented among selected features. The only cluster significantly overrepresented among the selected features was cluster 2 (p<0.05). Three out of five features of this cluster are also overrepresented individually in the full set of selected features – “Positive charge” (22 features), “Isoelectric point” (14 features) and “Normalized frequency of extended structure” (9 features) that describes the propensity of amino acids to form specific secondary structures [36]. Most of the selected features of this cluster describe residues in positions 319–320 and position 324 (Figure S7) and their relevance was confirmed by additional analysis described below.

**Figure 6:**
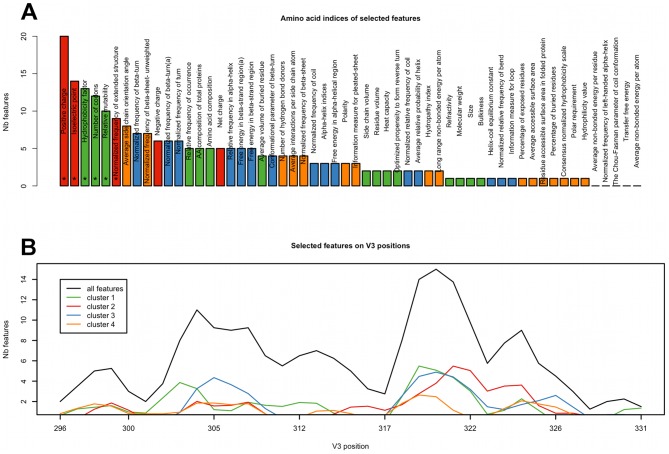
V3 positions and amino acid indices among the features of the clonal model. (A) Structure locations of features of the clonal model were mapped on the positions on the reference sequence. Numbers of selected features mapped to adjacent sequence positions were summed and averaged over a sequence window of size three. The resulting distribution of all features is represented by the black line, distributions of features of the four clusters are represented by lines in the relevant colors as defined in Figure 4. (B) Amino acid indices of the clonal model features. Bars are colored according to the clusters of amino acid indices. Significantly overrepresented indices are marked with an asterisk.

Next, we inspected which of the amino-acid indices most often appear among the selected features of the clonal model and analyzed the distribution of selected features along the V3 loop in a sliding-window approach (Figure 6). Among individual amino-acid indices we found six that are significantly enriched among selected features: “Positive charge”, “Isoelectric point”, “Hydrophobicity factor”, “Number of codons”, “Relative mutability” and “Normalized frequency of extended structure” (Figure 6A). Notably all of the significantly overrepresented indices belong to clusters 1 or 2. The sliding-window analysis of the distribution of selected features along the loop pointed to two regions: between positions 303 and 312 and more strongly between positions 318 and 324. These regions correspond to two strands of the V3 stem. In the first region (303–312) the selected features are based mostly on indices from clusters 1 and 3. The second region (318–324) shows also a high number of features based on indices from cluster 2 which are predominantly associated with residue charge (Figure 6B). A similar pattern of amino acid index overrepresentation and of feature distribution along the V3 loop was observed in the clinical model (Figure S8).

#### Structural regions

**Figure 7:**
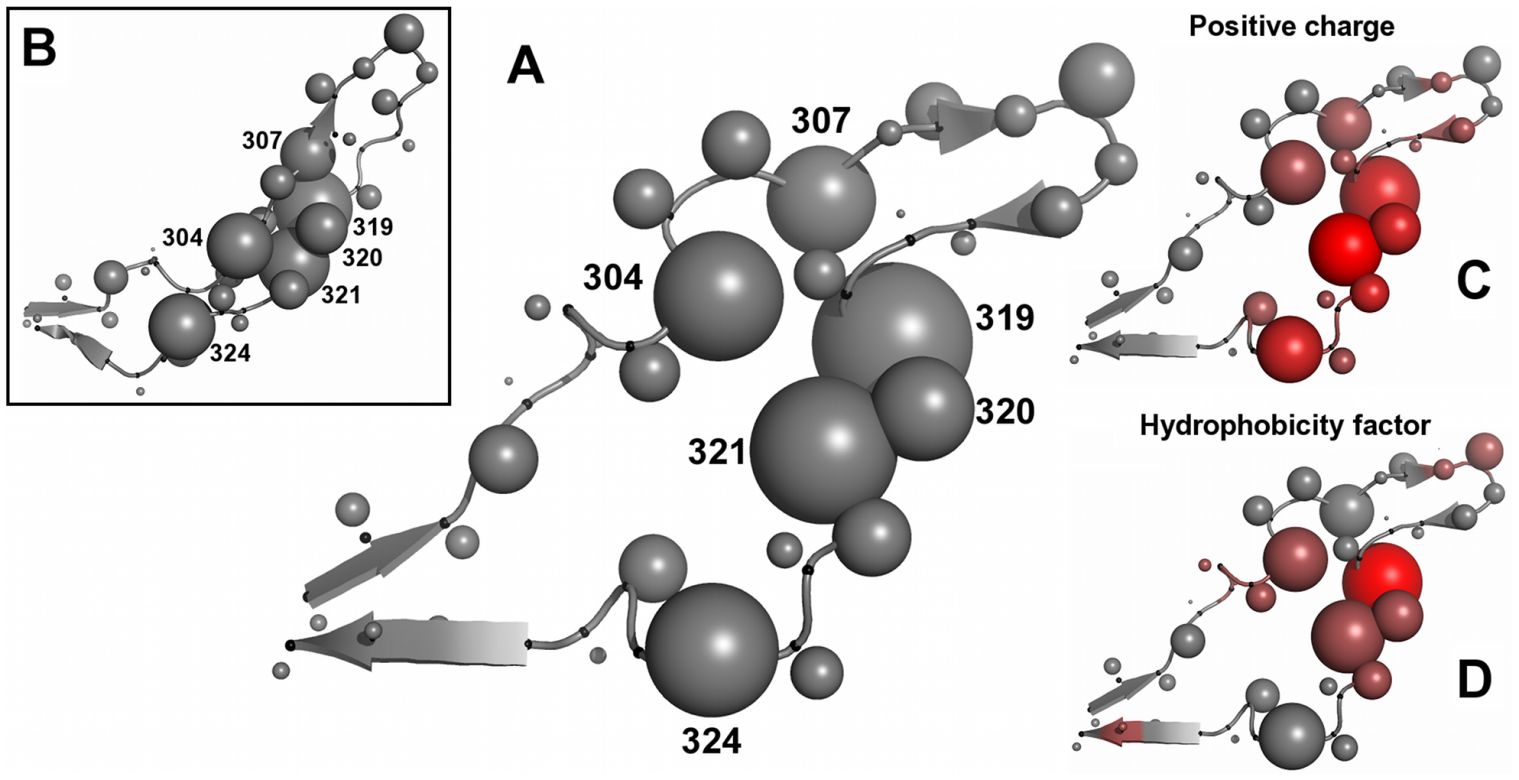
Important V3 positions and amino acid indices on the V3 structure. (A) 2B4C V3 structure used in this study. Cα atoms are marked with small black spheres along the loop backbone. Representative atoms are represented by gray spheres with the size proportional to the number of features of the clonal model mapped on the respective V3 position. Positions assigned to core sites are numbered. (B) V3 structure in a bound conformation (2QAD, [19]) with the same sphere representation as in panel (A). Positions informative of tropism are located close to each other in this conformation and form interactions between two sides of the loop stem. (C, D) Structure representation of V3 as in panel (A) with positions of the loop colored according to the ratio of selected features related to “Positive charge” (C) and “Hydrophobicity factor” (D) to the overall number of the selected features present on the respective V3 position with red indicating high ratio and gray low. Structures were visualized using Pymol [48].

To gain more insight into the two regions observed in the sliding-window test, we mapped the positions of the features of the clonal model on the crystal structure of the V3 loop (Figure 7). Most features of the two regions of interest describe positions 304, 307 and 319–321, respectively (Figure 7A). We label these regions *core site* (CS) 1 and 2. In the bound conformation of the loop (PDB code 2QAD) CS1 and CS2 are located closer to each other than in the open conformation (Figure 7B and Figure S9). We investigated the interactions of the residues of the two sites [37] and found that in the bound conformation residues of CS1 and CS2 form interacting pairs between two sides of the central loop stem. In particular residues 304 and 307, which are located on one side of the loop stem, form van-der-Waals interactions with residues 319 and 320, which are located on the other strand of the stem. In the open conformation CS1 and CS2 are more widely separated and the interactions between two sides of the loop are not observed. Position 324 is also associated with a high number of selected features and is located on the loop stem however does not interact with CSs in either of the conformations.

Features of the clonal model involving the six amino acid-indices that are significantly overrepresented among the selected features are all found in CS1 or CS2 as well as around residue 324 (see Figure 7 and Figure S10), which confirms the importance of CS1 and CS2 in determining coreceptor usage.

## Discussion

Physicochemical and structural properties of proteins determine their binding affinities. Prediction methods of HIV-1 coreceptor usage based solely on the V3 sequence do not account for this type of properties nor do they provide the information on loop characteristics that are crucial for the interaction. Prediction models incorporating loop structure can provide such information. However, previously reported structure-based prediction models suffer from limitations in terms of (i) runtime and software complexity – which prevents their accessibility via a tool publicly available online – and (ii) interpretation of the prediction result. Here we present a prediction model of HIV coreceptor usage based on V3 sequence and structure [18] that overcomes these limitations. The method is based upon a set of features that was selected from a large initial feature set. The model shows better performance than the one based on the initial feature set, both in terms of prediction accuracy and computational efficiency and shows higher predictive power than the prediction method based uniquely on sequence. In addition, the proposed model affords an interpretable set of physicochemical properties located in specific parts of the loop structure that play a role in determining viral tropism. The approach is generic and can be applied in other supervised learning applications involving the combination of sequence or evolutionary information, physicochemical and structural properties. In particular, computational biology and medical applications involving molecular binding mechanisms are good potential candidates for achieving improved accuracy and interpretability with the proposed approach.

Our clonal model was developed on a sequence set comprising different HIV-1 subtypes. The limited number of sequences of each subtype and the high variability of the V3 loop sequence which obviates a clear subtype classification advocate use of a common model for all subtypes, an approach also applied by other prediction methods [11], [14], [20], [21]. The proposed structural descriptor appears to entail information on both structure and sequence, as adding the binary encoding of the sequence to the descriptor does not improve the performance of the clonal model (Text S4). In contrast, the distance-based descriptor of Sander et al. [21] is complementary to the sequence which is demonstrated by the improved performance of the descriptor, when combined with the sequence information.

Our method shows a moderately but significantly higher prediction performance of approximately 3 percentage points over the model based on sequence only [14] both on the clonal and clinical datasets with and without patient clinical markers. The model additionally shows a higher or similar prediction performance to that of other structure-based methods [20], [21] without modeling steps that increase the computational cost of the prediction procedure. Our model shows higher prediction accuracy when applied on external datasets from these studies than on the dataset it was trained on, suggesting the selected feature set is not biased towards the used sequence set. Note that our approach implements an approximate representation of the structure of the V3 loop with the goal of predicting coreceptor usage based on an interpretable model and without predicting of an accurate structural model of the V3 loop with respect to insertions or deletions compared to the template structure. Also, unlike method of Sander et al. our approach does not involve modeling of side chains. The effectiveness of a thorough structural modeling and especially of modeling side chains for the purpose of predicting tropism is likely to be limited by the variability of the V3 loop sequence and the structural flexibility of the loop. In our method spheres are used to represent structural proximities over which physicochemical properties are averaged. In this way our approach accounts for the uncertainty of the structural conformation of the loop and avoids costly modeling steps. The accuracy and efficiency of our approach enables its use as a server application.

Unlike previously developed structure-based methods [20], [21], our method was tested not only on clonal data but also on clinically derived (bulk) data and showed significantly better performance over the established sequence-based approach. Given the common usage of this type of data in patient diagnosis and the potential difficulties it represents in classification [14], predicting tropism and MVC therapy outcome based on clinical data represents a more realistic scenario for training and assessment of classification methods than prediction of tropism based on clonal data.

We also assessed the capacity of our method to predict MVC therapy outcome. For the purpose of this validation, we used a cohort of patients treated with MVC. This analysis is limited due to the low number of cases in the MVC dataset. With increasing use of entry inhibitors, therapy outcome data are expected to become more abundant and the capacity to train models predicting therapy outcome will improve. The higher performance of the clinical over the clonal model in predicting therapy outcome suggests that comprehensive datasets appropriate for specific prediction goals can produce more reliable models.

The analysis of features informative of viral tropism points to two critical sites in the loop stem, comprising residues 304, 307 and 319–322, respectively and to position 324 located more closely to the base of the stem. The charge of amino acids at these sites is known to play role in coreceptor binding [6], [7]. Additionally, our analysis points to the importance of the propensities of these amino acids for forming specific secondary structures. Residues on both sides of the stem form interactions in the bound conformation of the loop probably contributing to the rigid form of the loop upon binding. The combined effect of charge and propensity for specific local structural conformations might therefore contribute to acquiring the adequate binding site complementarity and local loop conformation required for specific coreceptor binding.

The results of other studies of structural features related to HIV tropism are in general accordance with our results. A recently published method [38] predicts coreceptor usage based on a perturbation vector reflecting relative change in compatibility of a given V3 the sequence and structure with the reference structure [39]. Ten most important positions for the coreceptor usage, according to this study are positions 302–304, 306–307, 309, 312, 322, 324–325. However, no additional interpretation of the characteristics of these positions is provided. Sander et al. point to the residues 298, 302, 306, 308, 315, 317, 319, 321, 322 and 328 involved in residue pairs important for tropism. The regions found in our analysis are in close proximity or in-between the positions listed by Sander et al. on the V3 structure. However the ranking of Sander et al. is based on the importance of distances among functional atom types in the V3 loop, which is not equivalent to the importance of the residue itself. Findings reported by Dybowski et al. [20], point to electrostatic hulls around positions 306, 321 and 322, and between position 301 and 326 as the features of highest importance for the classification which is also in agreement with our results. Additionally, the authors point to hydrophobicity of residues 303 and 307 as important for viral tropism.

Given the considerable structural flexibility and sequence variability of the V3 loop, individual features of this region distinguishing between the two virus phenotypes are hard to define. We performed a comprehensive analysis of a large number of physicochemical residue characteristics in various locations on the loop and pointed to those that are the most informative of tropism. The resulting method offers higher performance than the standard sequence-based approach with a comparable efficiency and a direct interpretation of structural and physicochemical determinants of tropism. The method has been implemented as a server application within the geno2pheno framework under http://structure.bioinf.mpi-inf.mpg.de/.

## Materials and Methods

### Dataset

To construct the clonal dataset we screened the Los Alamos database [25] for all phenotyped V3 loop sequences. In order to avoid bias due to overrepresentation of data from the same patient we filtered the dataset extracting one randomly chosen sequence per patient. The resulting dataset contains 1186 sequences with tropism annotation, 215 of which are annotated as X4 viruses. In the dataset 501 sequences are of subtype B, 286 of subtype C, the remaining sequences are of other and recombinant subtypes. We aligned the sequences in the dataset and the sequence of the V3 loop of the PDB entry 2B4C using ClustalW [40] obtaining an alignment of length 50. In order to assess the robustness of this alignment we aligned each of the sequences in our dataset to the alignment of all the remaining sequences. In this test all the alignments were identically reproduced suggesting that the sequence alignment prior to prediction results in the correct alignment. The clonal dataset is provided as Supplemental Text S7. In our study the positions in the gp120 sequence are numbered relative to the reference as previously described [41]. See Figure S11 for the correspondence between the numbering of the V3 loop positions in the reference sequence HXBc2 [41] and the subtype B consensus sequence.

### Amino-acid indices

We used the amino-acid indices collected in the AAindex database [23]. In this database various physicochemical and biochemical properties of amino acids are stored in the form of numerical indices. Due to the high number of over 500 indices in the database many of which are redundant we used a representative and interpretable subset of 54 indices, selected using multivariate statistical analysis [24]. This is a minimal fully representative set of indices. Reducing it further would limit the physicochemical information provided by our descriptor. Two of the selected indices named “Normalized frequency of beta-turn” and “Free energy in beta-strand region” were represented by duplicate entries in the AAindex database showing minor differences (AAindex entries: CHOP780101/CHOP780203 and MUNV940104/MUNV940105 respectively). To avoid arbitrary selection between the duplicate entries we used both of the ambiguous indices, which resulted in a set of 56 indices selected for this study.

### Structural descriptor

The descriptor of the V3 loop was based on the published structure of the V3 loop with PDB [42] code 2B4C [18]. To construct the descriptor for each V3 loop sequence we used *spheres* defining structural neighbors inside the loop structure within which the physicochemical properties of residues are averaged as detailed below. The spheres are positioned along the reference loop backbone and centered at its residues. Specifically, positions of residues were defined as the position of the *representative atom* of each residue in the structure – the Cα atom for Glycine and the Cβ atom for all other amino acid types. Positions of insertions in the alignment relative to the reference structure were inferred based on the positions of representative atoms of the residues at both ends of the insertions (*flanking atoms*). First, a line connecting the flanking atoms was calculated. Then the inserted residues were placed along the line at equidistant positions. This way we approximate the location of atoms on the loop structure without precise modeling of the structure which is likely to be inaccurate given the flexibility of the V3 loop structure and which would considerably slow down the prediction process. The resulting coordinates of the residues of the V3 loop sequences were used as centers of the spheres defining the structural neighborhoods in the loop structure.

In addition to the set of spheres corresponding to alignment positions additional spheres were positioned at the midpoints of lines connecting centers of each pair of consecutive alignment spheres. This way we obtained a set of 99 spheres – 50 corresponding to alignment positions and 49 positioned in-between consecutive alignment positions. Example spheres are illustrated in Figure 1.

Each V3 sequence position was mapped to a sphere if the corresponding representative atom was located within the given sphere. The details of the selection of the sphere radius and Gaussian smoothing parameter within the spheres are described and illustrated in Text S1 and Figure S1.

### Prediction method

The model based on the structural descriptor classifying viruses as R5 or X4 was constructed using a linear SVM [43] implemented in the R package e1071 [44]. For model evaluation we used the ROC curve that illustrates the trade-off between specificity and sensitivity. The AUC and the specificity at the sensitivity of the 11/25 rule were used as measures of model performance. We used the R package ROCR [45] for visualization and evaluated the models with ten times ten (10×10) fold cross validation. Each descriptor feature was normalized to [0,1] within the training dataset.

### Feature selection

We used two classification methods performing feature ranking: Random Forests (RF) [26], with the mean decrease in Gini index and linear SVMs with the feature weights as two measures of feature importance [27]. We also used Lasso regression [28] which performs feature selection by assigning zero coefficients to the less important features. For the methods producing feature ranking (RF and linear SVM) we tested two cutoffs for the selected features: top 1% and top 5% of a gamma distribution fitted to the ranking of all the features using maximum likelihood. We used all features selected by the Lasso regression method. The feature ranking of the SVM and Lasso regression methods was obtained via an average of a 10×10-fold cross validation. The RF method performs internal randomization, its feature ranking was therefore inferred from a single run of the method. We tested the performance of models based on subsets of features selected by each method and combined feature sets selected by different methods. Models based on subsets of selected features were named after the feature selection method with the percent cutoff indicated in parentheses (e.g. SVM(1)). Names of models based on combinations of feature sets selected using several feature selection methods were composed of the corresponding feature selection methods separated by an underscore (e.g. SVM(1)_Lasso). As the analysis of the features selected for the clonal model was a goal of our study, feature selection was performed on the entire clonal dataset. To assess how the choice of the set of sequences on which the features are selected impacts the model's prediction accuracy, we performed two different types of tests. In the first test, features of the model were reselected on the training set in each cross validation run on the clonal set (nested cross validation). In the second test we applied the features of the clonal model to other sequence sets – Sander and Dybowski datasets.

### Other datasets

The HOMER dataset was filtered to contain one randomly chosen sequence per patient, which resulted in a set of 954 sequences out of which 167 comprised X4 viruses. Each sequence in the clinical dataset represents a population of variants genotyped and phenotyped in bulk, an approach used in the routine clinical practice. These sequences contain ambiguous positions with alternative amino acids representing different variants in the population. The ambiguous positions were represented by a balanced average of vectors of indices of all alternative amino acids at a given position. Due to these differences between the clinically and clonally derived data, we repeated the feature selection on this dataset and constructed the *clinical model*.

The MVC dataset comprises 53 patient cases under MVC therapy whose therapy outcome can be assessed based on the viral load (VL). We define as therapy success an observed 2log decrease in VL with respect to the level immediately before the therapy start or a VL drop below 50 copies/ml measured three months after the therapy start [46]. We classified the viruses sequenced at therapy start with respect to their tropism in order to investigate the capacity of the structural descriptor to predict the therapy outcome. Since the MVC dataset was derived in clinical bulk sequencing we used the clinical model to predict the phenotype of the sequences in this dataset. We used the prediction score at the specificity of 11/25 rule which corresponds to a false discovery rate (FDR) of 6.28% in the HOMER dataset as a classification cutoff between the R5 and X4 viruses. The FDR is an estimate of the expected proportion of sequences incorrectly classified as X4 viruses with a given cutoff and is calculated as the fraction of R5 viruses in the training set scored above the cutoff among all sequences scored above the cutoff in 10×10-fold cross validation. In the MVC dataset we additionally distinguish 28 sequences that were phenotyped using the Trofile assay (*Trofile* dataset). Summary statistics for all datasets used are presented in Table 3.

**Table 3:** Summary statistic of the used datasets.

dataset	all sequences	X4 sequences	R5 sequences
clonal	1188	215	973
Sander	1357	205	1152
Dybowski	515	151	364
MVC*	53	5	48
Trofile	28	3	25

* For the MVC dataset in the columns “X4 sequences” and “R5 sequences” the numbers of therapy failures and successes are shown respectively.

### Feature clustering

Clustering of the 56 amino acid indices was performed in order to facilitate the interpretation of the large number of selected features. As a similarity score among the indices we used the absolute value of their correlation. This way, indices that express the same affinities among amino acids are considered similar. We performed hierarchical clustering of the 56 amino acid indices and computed silhouette values [47] in order to select the best set of clusters. The highest silhouette value was obtained for a partitioning of indices into 12 clusters. The highest silhouette value of a partitioning of indices into fewer than 12 clusters was obtained for four clusters. We selected four as the number of clusters for further analysis as it represents a small and interpretable number of groups of indices. The silhouette values as well as the 12 clusters are shown in Figures S12 and S13.

## Supporting Information

Figure S1 Choice of sphere radius and Gaussian smoothing parameters. Black histograms represent the distribution of the number of residues included in proximities of a radius indicated on the corresponding plot on the left. Red histograms illustrate the sum of Gaussian normalizing factor per each residue. Mean with variance in brackets of each distribution are indicated in legends.
(TIFF)

Figure S2 Performance of models based of structural proximities of different radii. ROCR of models based on different radii are plotted. The selected radius of 8 Å is traced with a black solid line. AUC and sensitivity at the specificity of 11/25 rule in brackets are indicated in the legend.
(TIFF)

Figure S3 Distance on the 3D structure of features selected by different feature selection methods. Plot in D illustrates the overall distance of spheres of the features of the initial feature set. Plots in A–C illustrate the distance of the highly correlated features as defined above. The highly correlated features can be found among features selected by different methods and they pertain to locations in close proximity on the structure, which is the potential reason for the low overlap of features selected by different methods.
(TIFF)

Figure S4 Correlation of features in the initial feature set. Histograms show distribution of the Pearson correlation of all features of the structural descriptor (left panel), of the features of the clonal model (middle panel) and of the features of the clonal model with the remaining features of the structural descriptor (right panel). Median, percentage of feature pair with correlation >0.5 and >0.75 are indicated in the legend.
(TIFF)

Figure S5 Comparison of the clonal and g2p models in the precision-recall space. The curves show the relationship between true positive rate (recall) and positive predictive value (precision). Area under the curve shows a higher predictive performance of the clonal model compared to the g2p model.
(TIFF)

Figure S6 Validation of the clinical model on an external dataset. In order to support the assessment of the performance of the clinical model we used an independent dataset of 760 clinically-derived sequences phenotyped using Enhanced (140 sequences) and standard Trofile (620 sequences). The clinical model shows a visibly better performance compared to the clonal model on the sequences phenotyped using the enhanced Trofile assay (left panel, solid black and red curve, respectively) and outperforms g2p model train on clinical or clonal data (left panel, dashed black and red curve, respectively). These differences in performance between the clinical and clonal models are not observed on the subset of sequences phenotyped with the standard Trofile assay (right panel). Nevertheless, in this subset, the structure-based models outperform the corresponding sequence-based models by <2 percentage points.
(TIFF)

Figure S7 Effect of indels on the prediction accuracy of the clonal model. The curves illustrate the prediction performance of the clonal model based on a dataset containing only sequences with indels (black curve) and only sequences with indels (red curve). Similar performance of the clonal model based on both datasets suggests there is a limited effect of the presence of indels on the model accuracy.
(TIF)

Figure S8 Distribution of V3 positions (top panel) and amino acid indices (bottom panel) among the features selected for the clinical model constructed analogous to Figure 6 in the main text. Clinical model is composed of a lower number of features (66) compared to clonal model. Although two regions corresponding to CS1 and CS2 are discernable (top panel), they are generally more spread out. This might be due to a lower number of features in this model and higher variability of sequences in the clinical dataset. Similar to the features of the clonal model the significantly overrepresented amino acid indices in the clinical model belong mainly to cluster 1 and 2 (bottom panel) and relate to residue charge and hydrophobicity.
(TIFF)

Figure S9 Side-chains of the V3 loop in the unbound (A, structure 2B4C) and bound (B, structure 2QAD) conformation. In the bound conformation the residues of CS1 (304 and 307) and CS2 (319–321) are closely located and form bonds between two sides of the loop stem.
(TIF)

Figure S10 Clusters of selected features mapped on the 2B4C structure.
(TIF)

Figure S11 Significantly overrepresented features mapped on the 2B4C structure.
(TIFF)

Figure S12 V3 residue numbering. The numbering of V3 residues used in this manuscript is shown on the 2B4C structure. Top numbers indicate residue position within V3 loop, bottom numbers are assigned according to HXBc2, a numbering used also in the 2B4C annotation [18]. Figure from [21].
(TIFF)

Figure S13 Hierarchical clustering of amino acid indices. Black dots indicate numbers of clusters, red dots the silhouette values for the consecutive steps of the clustering procedure. Vertical lines indicate the best clustering obtained for 12 clusters and second best with a lower number of clusters (4).
(TIFF)

Figure S14 Separation of amino acid indices into 12 clusters – the separation that showed the largest silhouette value.
(TIFF)

Table S1 Performance of the clinical model and models derived from the clinical dataset by removing sequences with ambiguities (HOMER-filter), removing sequences without ambiguities (HOMER-ambi) and replacing ambiguities with gaps (HOMER-gap).
(PDF)

Table S2 Therapy outcome prediction using structure-based model.
(XLS)

Table S3 Therapy outcome prediction using g2p model.
(XLS)

Table S4 Ranking and selection of features.
(XLS)

Table S5 AA indices of the selected features.
(XLS)

Table S6 Positions of the V3 loop of the selected features.
(XLS)

Text S1 Selection of model parameters.
(PDF)

Text S2 Overlap of features selected by different feature selection methods.
(PDF)

Text S3 Feature correlation.
(PDF)

Text S4 Combining structure and sequence descriptors.
(PDF)

Text S5 Validation of the clinical model on external dataset.
(PDF)

Text S6 Effect of ambiguities on prediction accuracy.
(PDF)

Text S7 Clonal dataset.
(ZIP)
