# Supplementary figures and images for: Analysis of Physicochemical and Structural Properties Determining HIV-1 Coreceptor Usage
Source: PLoS Comput Biol. 2013 Mar 21;9(3):e1002977. doi: 10.1371/journal.pcbi.1002977 (PMC3605109; doi:10.1371/journal.pcbi.1002977)

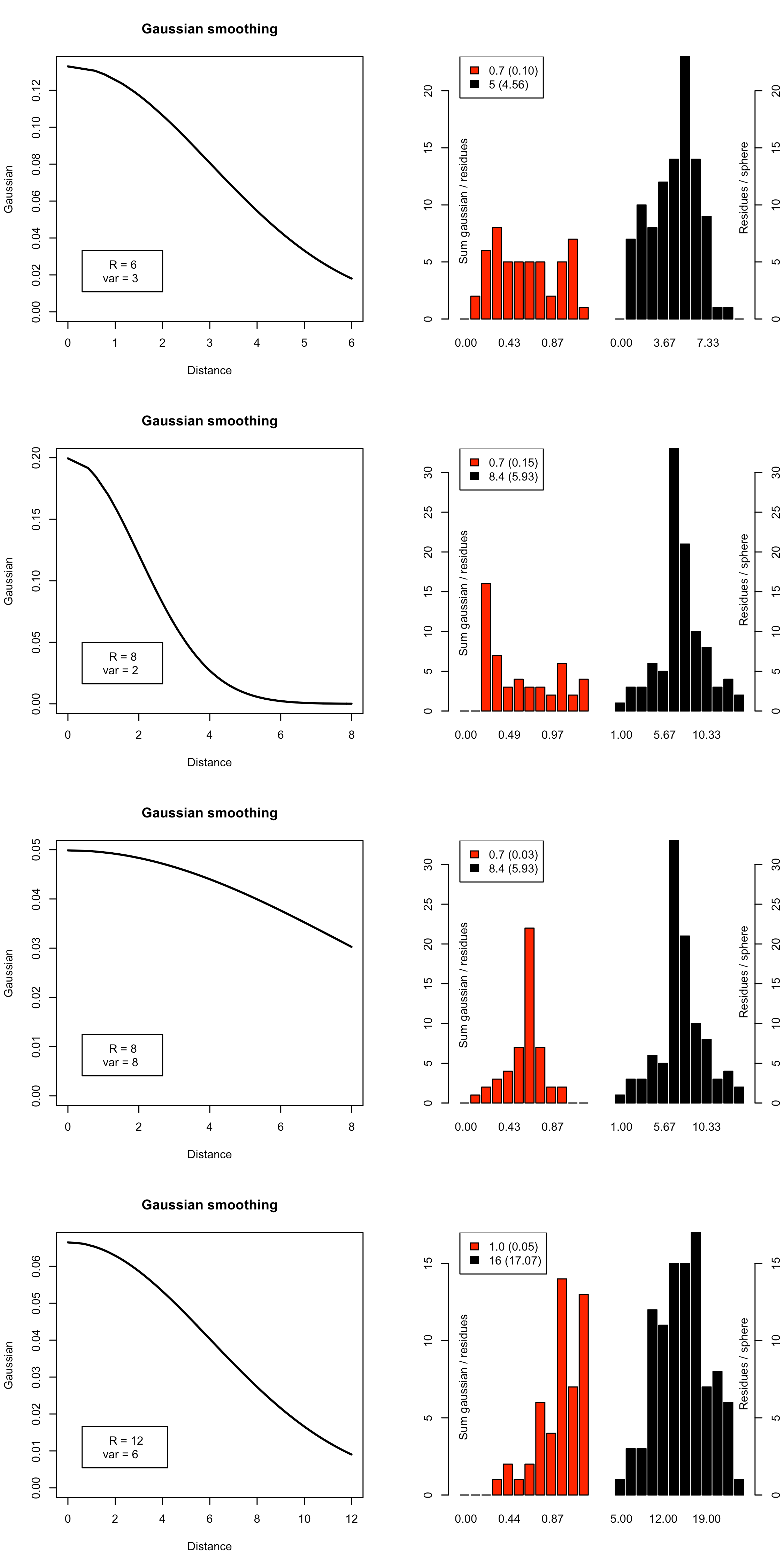

Supplement: Figure S1 — Choice of sphere radius and Gaussian smoothing parameters. Black histograms represent the distribution of the number of residues included in proximities of a radius indicated on the corresponding plot on the left. Red histograms illustrate the sum of Gaussian normalizing factor per each residue. Mean with variance in brackets of each distribution are indicated in legends. (TIFF) [file pcbi.1002977.s001.tif]

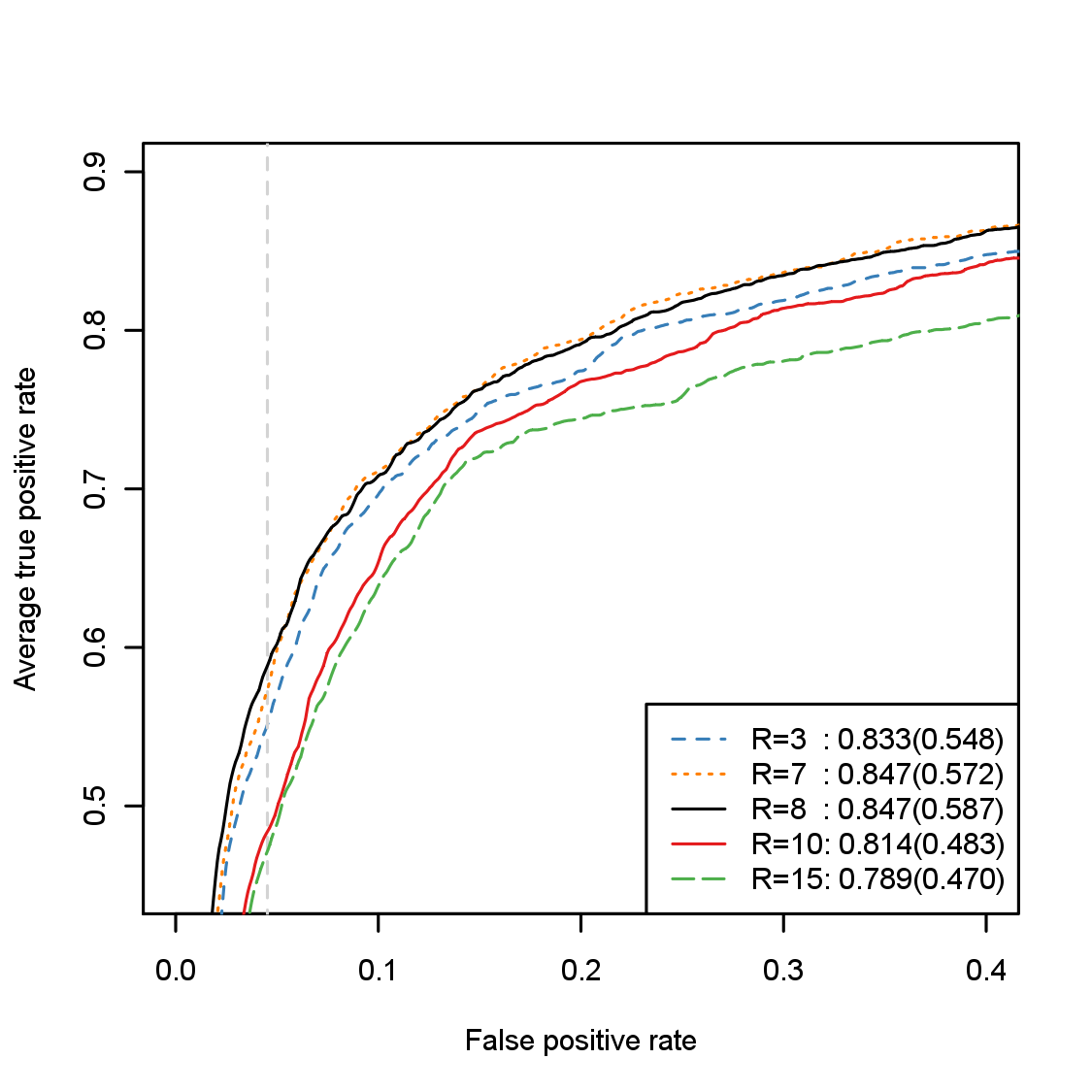

Supplement: Figure S2 — Performance of models based of structural proximities of different radii. ROCR of models based on different radii are plotted. The selected radius of 8 Å is traced with a black solid line. AUC and sensitivity at the specificity of 11/25 rule in brackets are indicated in the legend. (TIFF) [file pcbi.1002977.s002.tif]

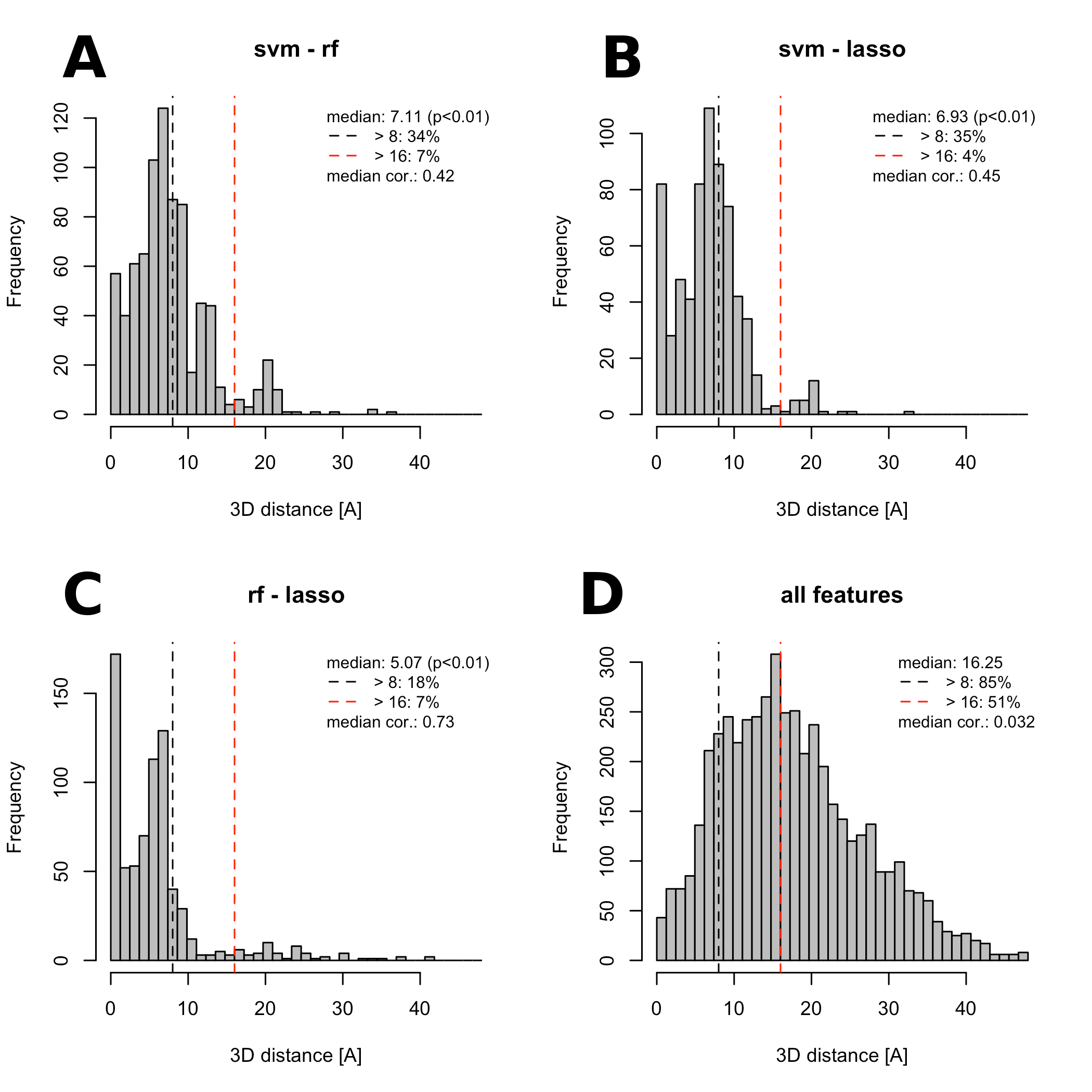

Supplement: Figure S3 — Distance on the 3D structure of features selected by different feature selection methods. Plot in D illustrates the overall distance of spheres of the features of the initial feature set. Plots in A–C illustrate the distance of the highly correlated features as defined above. The highly correlated features can be found among features selected by different methods and they pertain to locations in close proximity on the structure, which is the potential reason for the low overlap of features selected by different methods. (TIFF) [file pcbi.1002977.s003.tif]

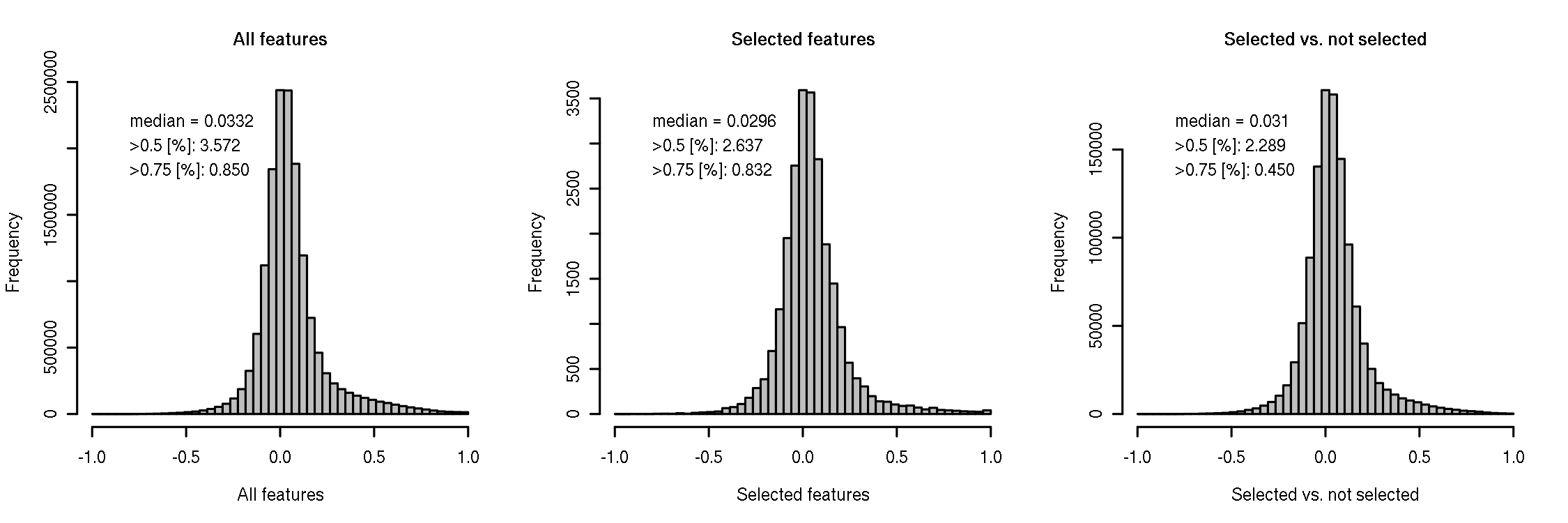

Supplement: Figure S4 — Correlation of features in the initial feature set. Histograms show distribution of the Pearson correlation of all features of the structural descriptor (left panel), of the features of the clonal model (middle panel) and of the features of the clonal model with the remaining features of the structural descriptor (right panel). Median, percentage of feature pair with correlation >0.5 and >0.75 are indicated in the legend. (TIFF) [file pcbi.1002977.s004.tif]

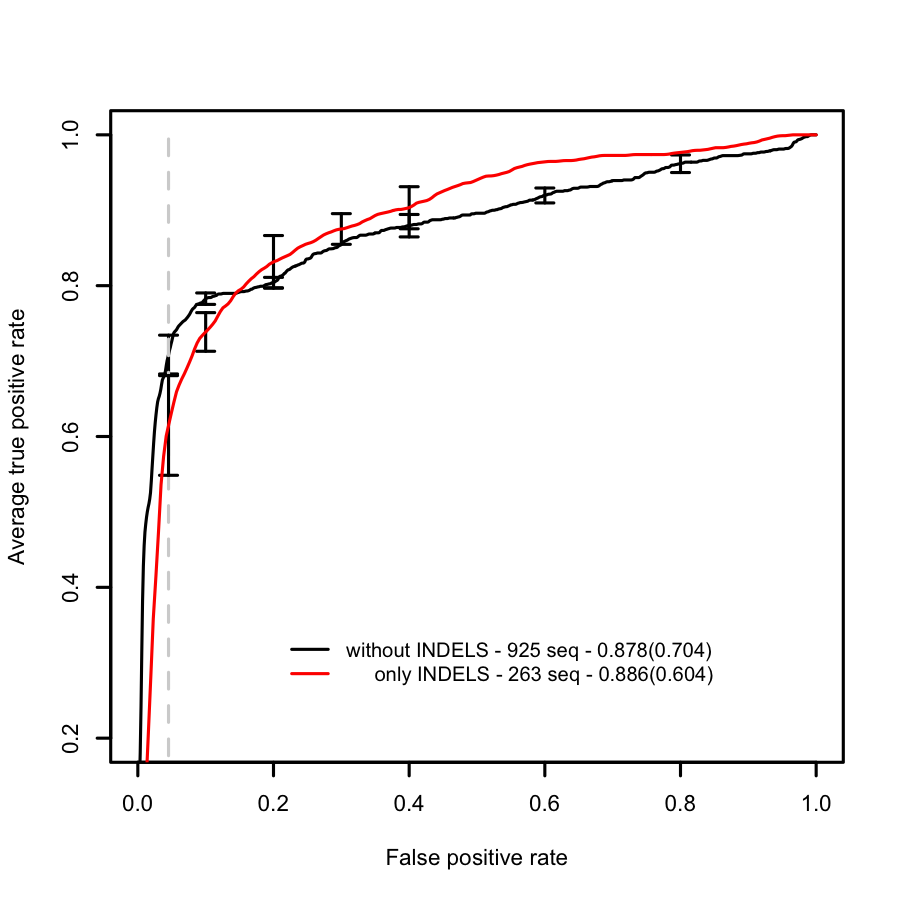

Supplement: Figure S5 — Comparison of the clonal and g2p models in the precision-recall space. The curves show the relationship between true positive rate (recall) and positive predictive value (precision). Area under the curve shows a higher predictive performance of the clonal model compared to the g2p model. (TIFF) [file pcbi.1002977.s005.tif]

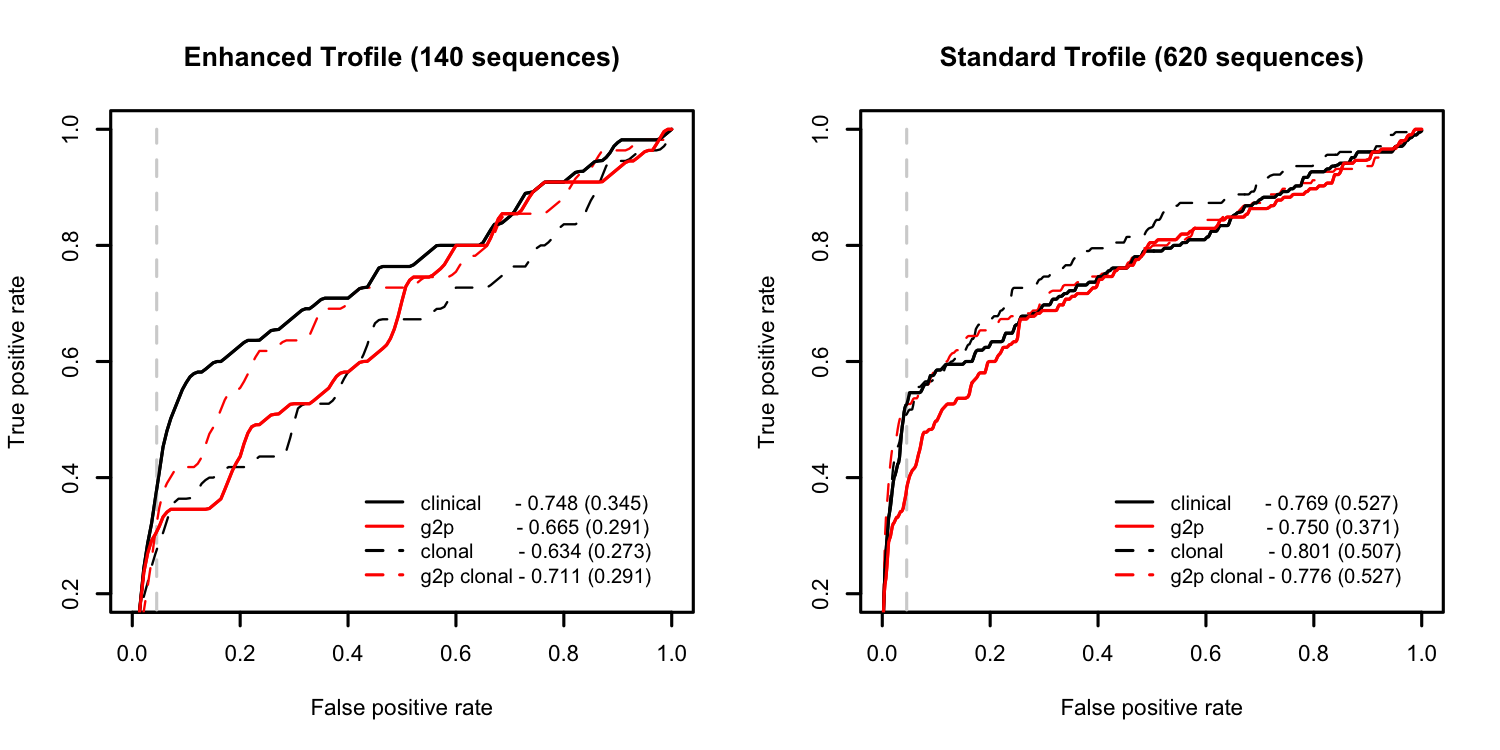

Supplement: Figure S6 — Validation of the clinical model on an external dataset. In order to support the assessment of the performance of the clinical model we used an independent dataset of 760 clinically-derived sequences phenotyped using Enhanced (140 sequences) and standard Trofile (620 sequences). The clinical model shows a visibly better performance compared to the clonal model on the sequences phenotyped using the enhanced Trofile assay (left panel, solid black and red curve, respectively) and outperforms g2p model train on clinical or clonal data (left panel, dashed black and red curve, respectively). These differences in performance between the clinical and clonal models are not observed on the subset of sequences phenotyped with the standard Trofile assay (right panel). Nevertheless, in this subset, the structure-based models outperform the corresponding sequence-based models by <2 percentage points. (TIFF) [file pcbi.1002977.s006.tif]

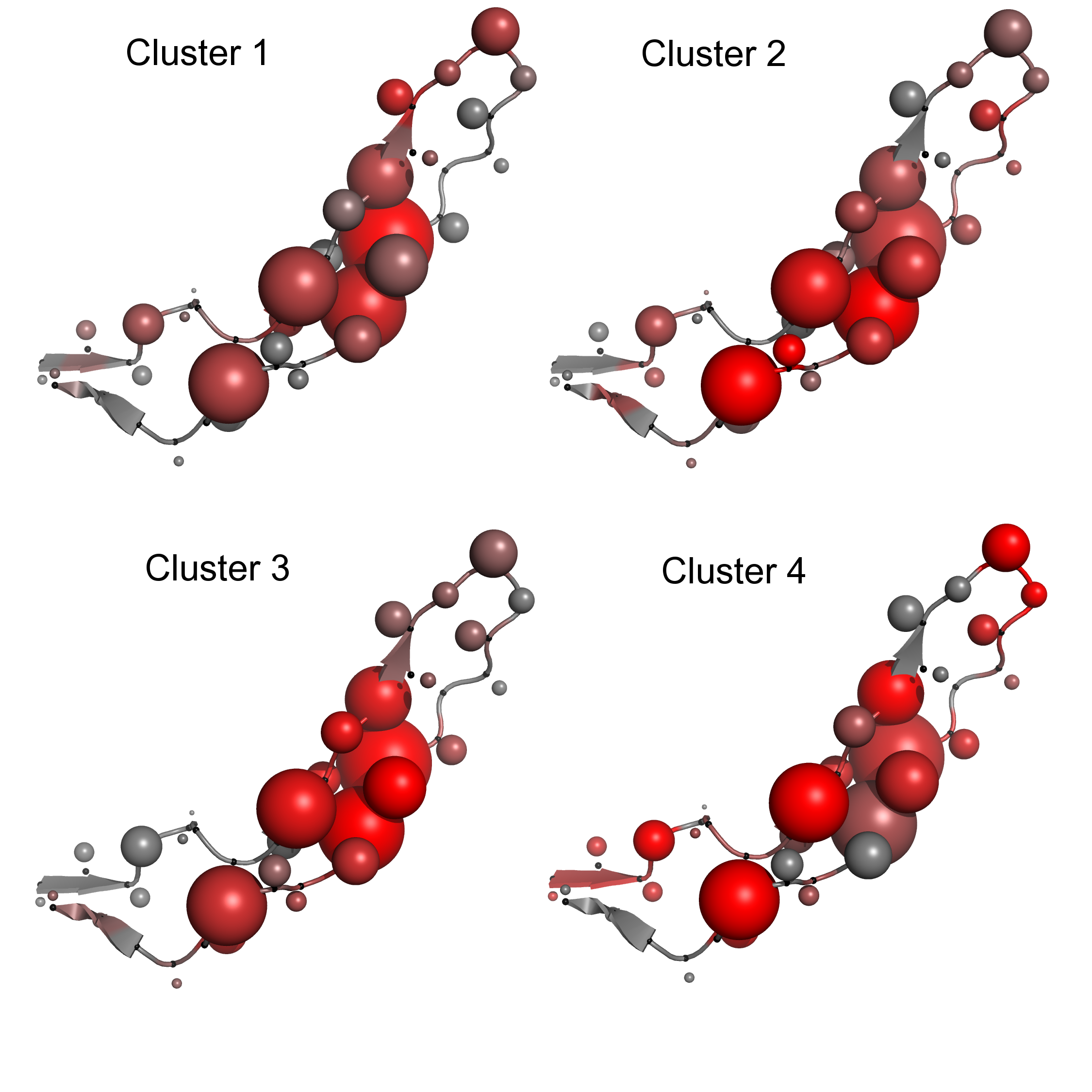

Supplement: Figure S7 — Effect of indels on the prediction accuracy of the clonal model. The curves illustrate the prediction performance of the clonal model based on a dataset containing only sequences with indels (black curve) and only sequences with indels (red curve). Similar performance of the clonal model based on both datasets suggests there is a limited effect of the presence of indels on the model accuracy. (TIF) [file pcbi.1002977.s007.tif]

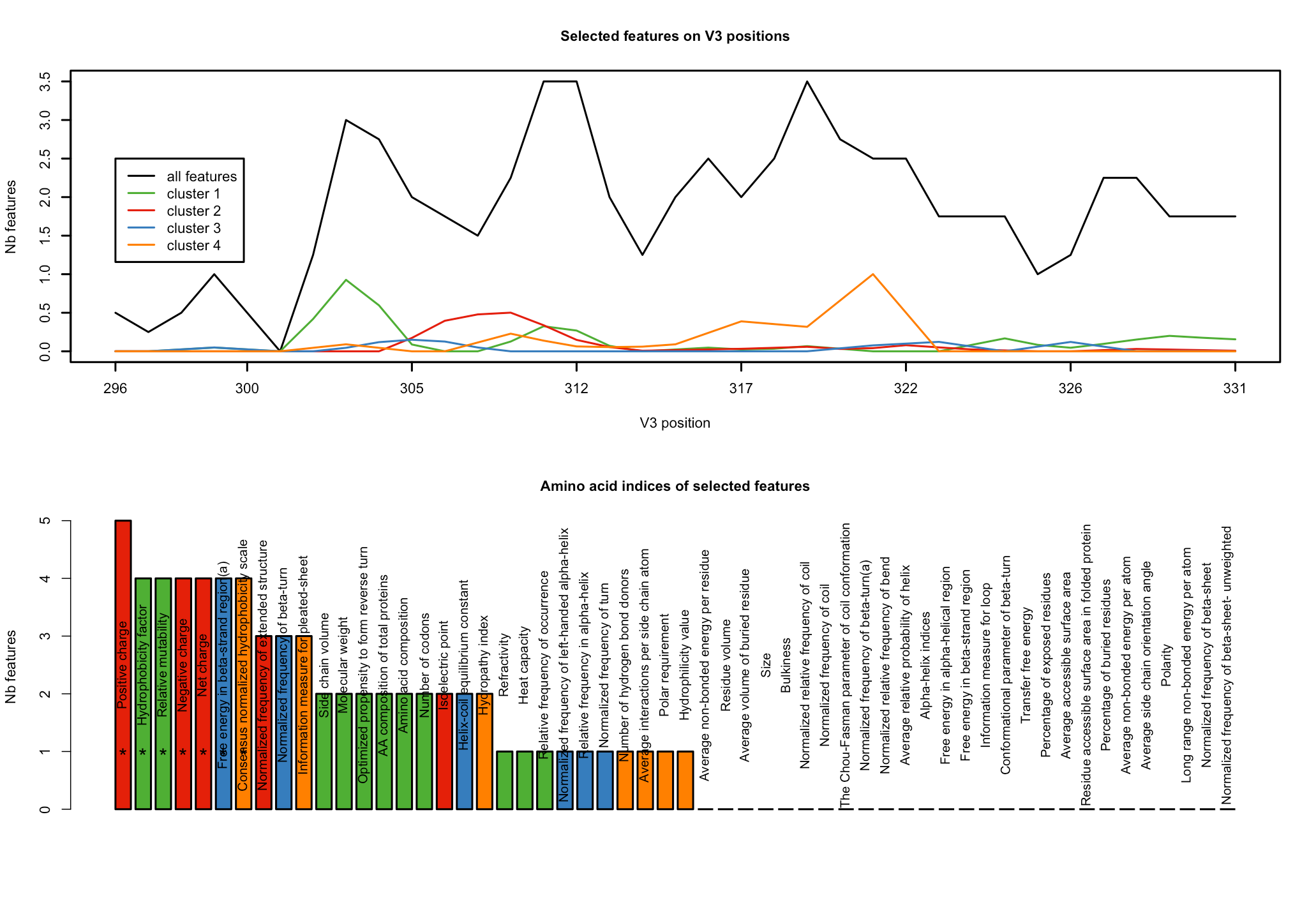

Supplement: Figure S8 — Distribution of V3 positions (top panel) and amino acid indices (bottom panel) among the features selected for the clinical model constructed analogous to Figure 6 in the main text. Clinical model is composed of a lower number of features (66) compared to clonal model. Although two regions corresponding to CS1 and CS2 are discernable (top panel), they are generally more spread out. This might be due to a lower number of features in this model and higher variability of sequences in the clinical dataset. Similar to the features of the clonal model the significantly overrepresented amino acid indices in the clinical model belong mainly to cluster 1 and 2 (bottom panel) and relate to residue charge and hydrophobicity. (TIFF) [file pcbi.1002977.s008.tif]

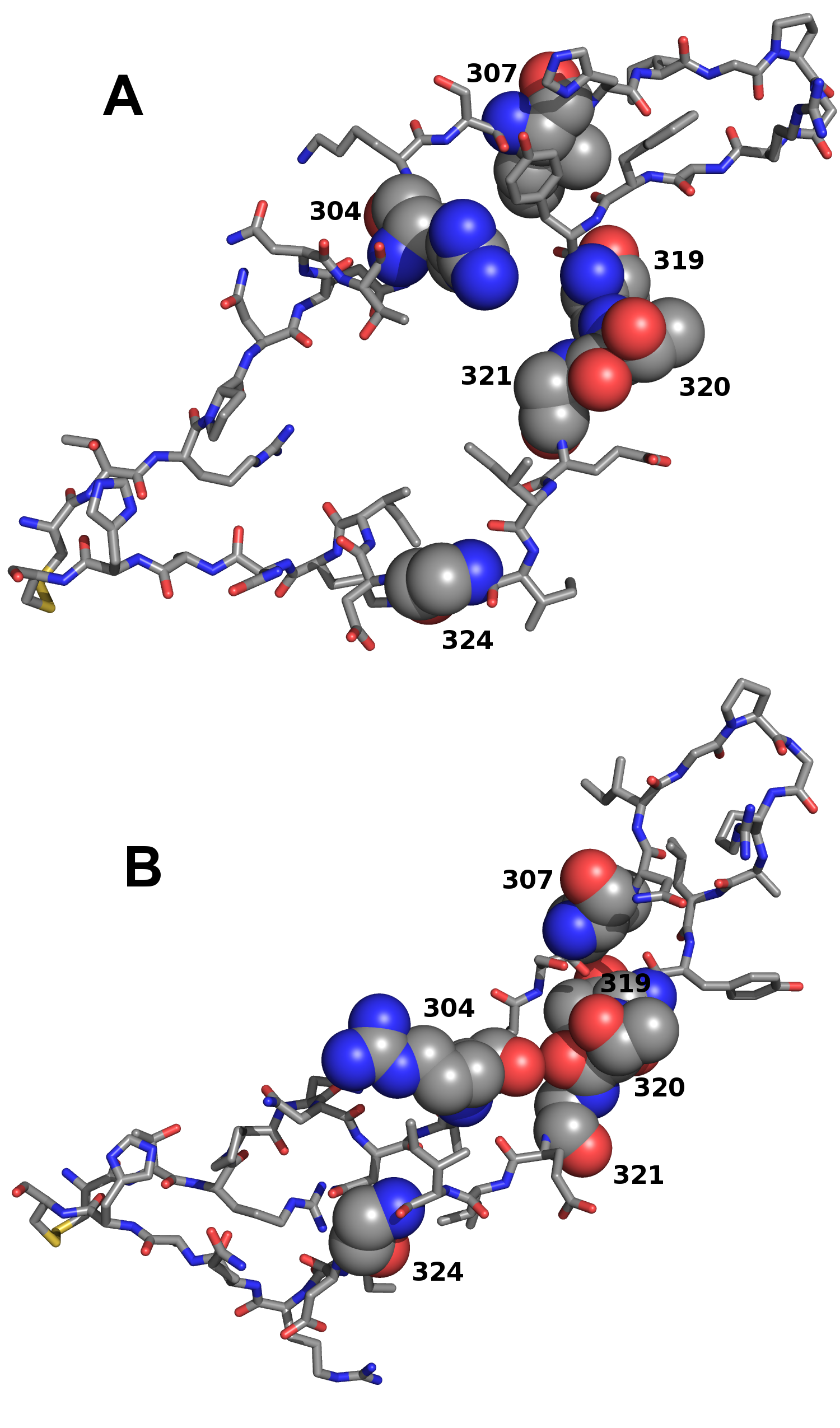

Supplement: Figure S9 — Side-chains of the V3 loop in the unbound (A, structure 2B4C) and bound (B, structure 2QAD) conformation. In the bound conformation the residues of CS1 (304 and 307) and CS2 (319–321) are closely located and form bonds between two sides of the loop stem. (TIF) [file pcbi.1002977.s009.tif]

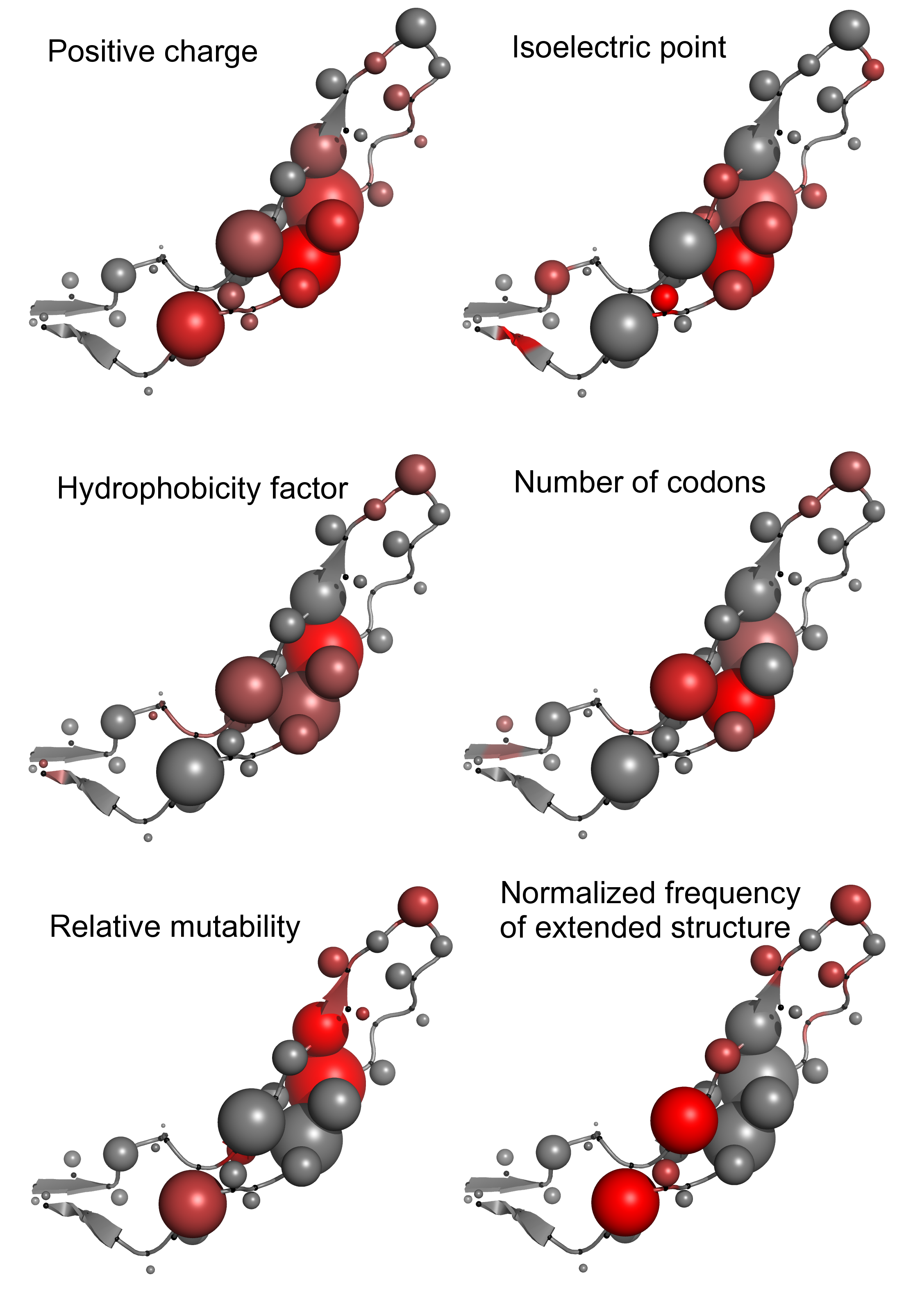

Supplement: Figure S10 — Clusters of selected features mapped on the 2B4C structure. (TIF) [file pcbi.1002977.s010.tif]

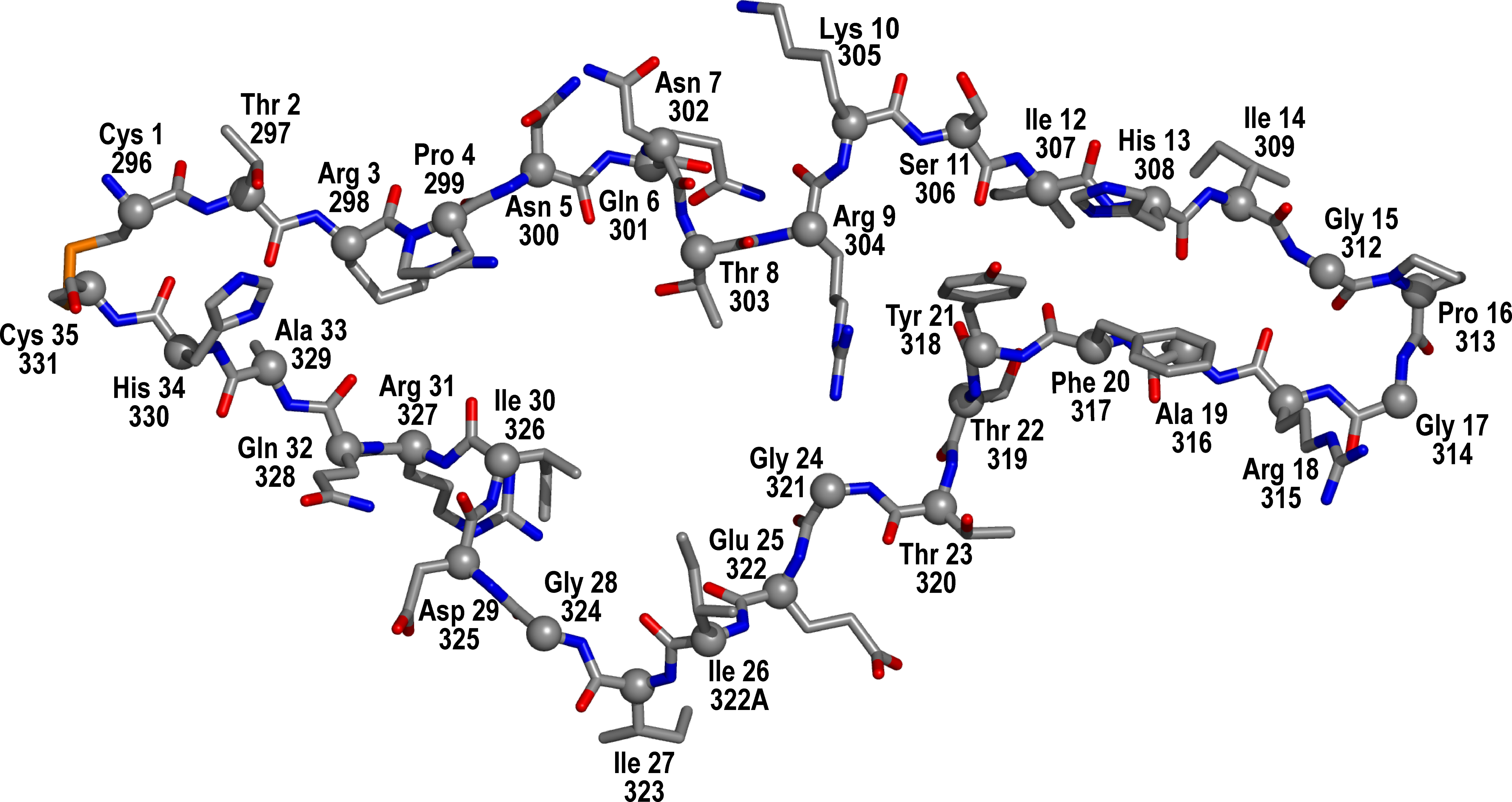

Supplement: Figure S11 — Significantly overrepresented features mapped on the 2B4C structure. (TIFF) [file pcbi.1002977.s011.tif]

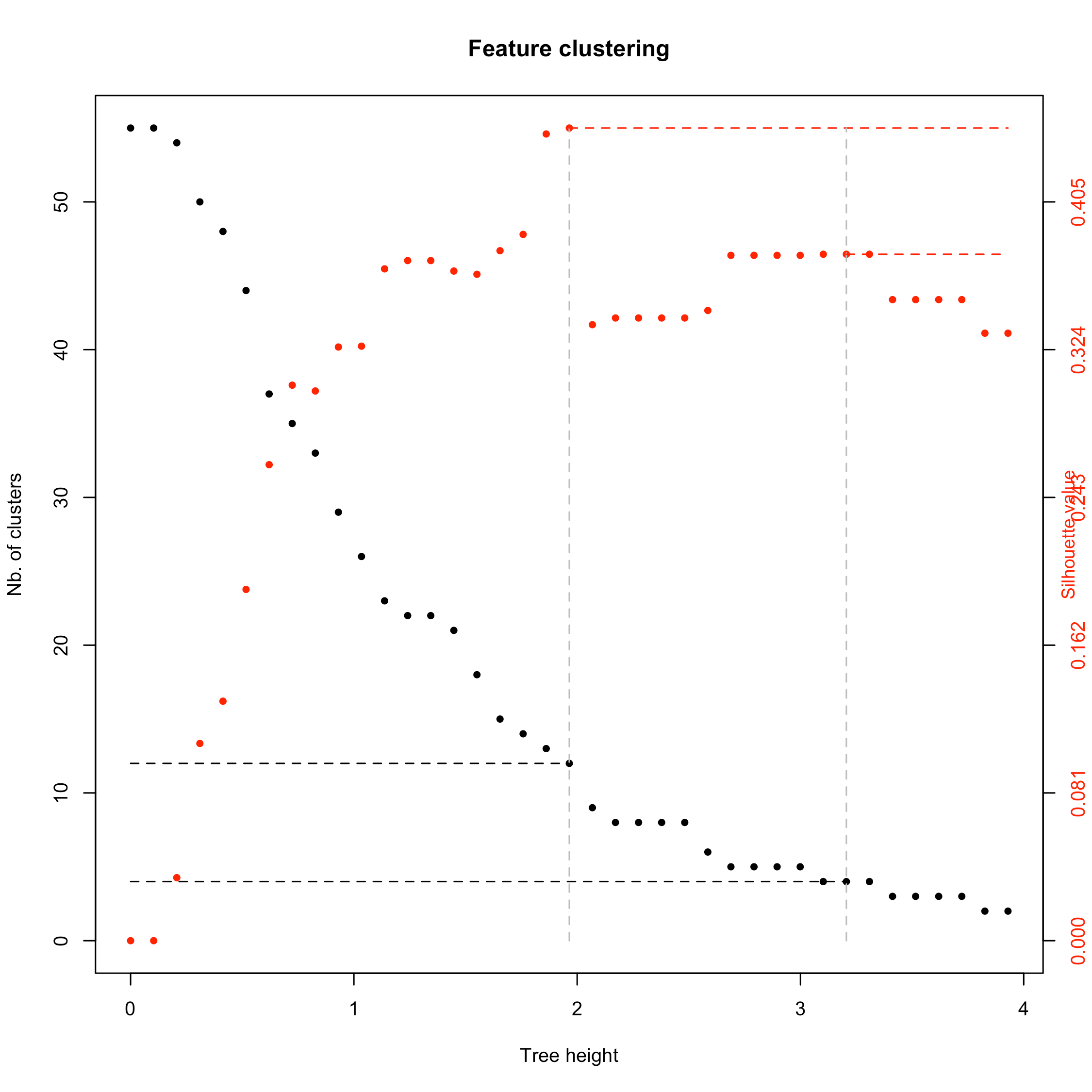

Supplement: Figure S12 — V3 residue numbering. The numbering of V3 residues used in this manuscript is shown on the 2B4C structure. Top numbers indicate residue position within V3 loop, bottom numbers are assigned according to HXBc2, a numbering used also in the 2B4C annotation [18]. Figure from [21]. (TIFF) [file pcbi.1002977.s012.tif]

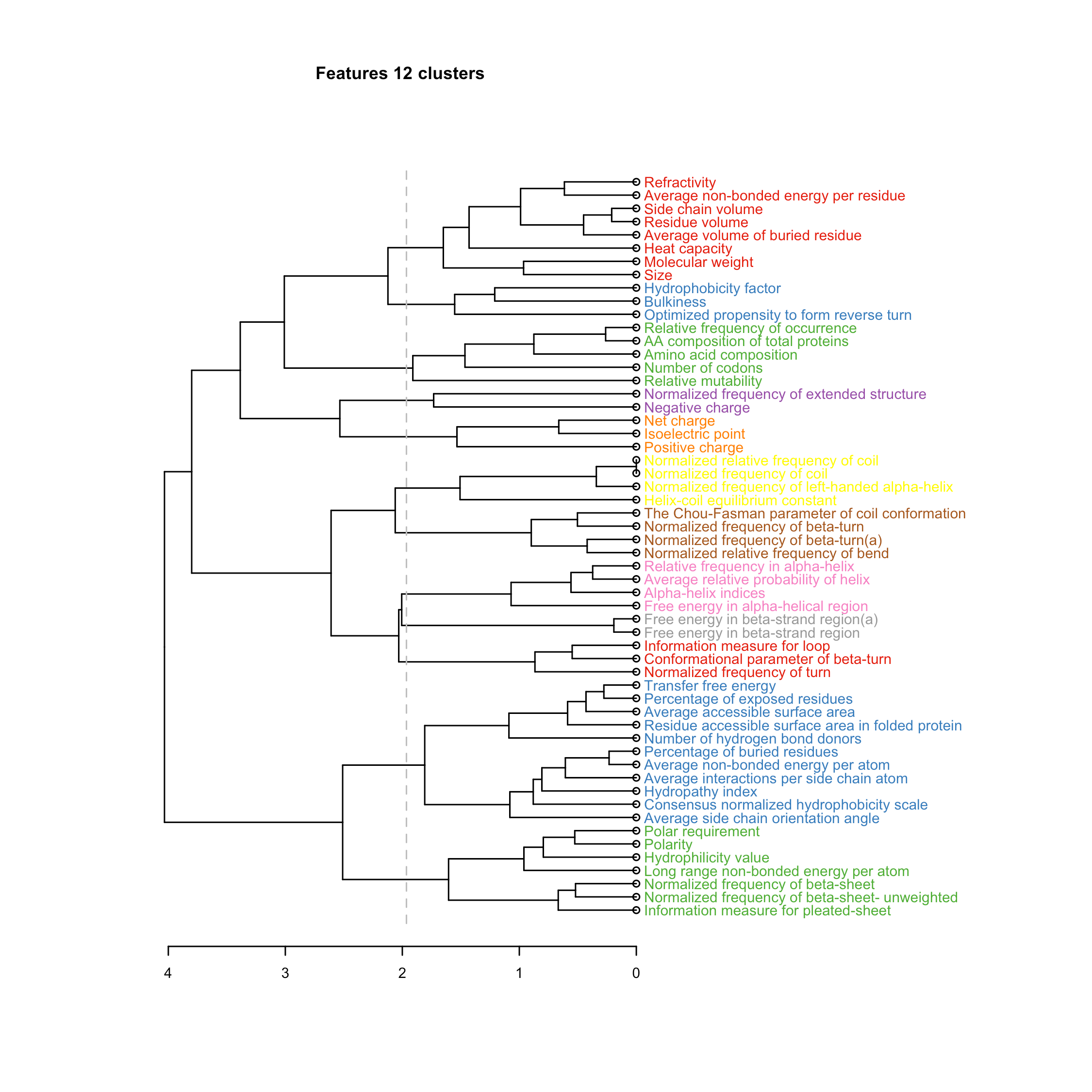

Supplement: Figure S13 — Hierarchical clustering of amino acid indices. Black dots indicate numbers of clusters, red dots the silhouette values for the consecutive steps of the clustering procedure. Vertical lines indicate the best clustering obtained for 12 clusters and second best with a lower number of clusters (4). (TIFF) [file pcbi.1002977.s013.tif]

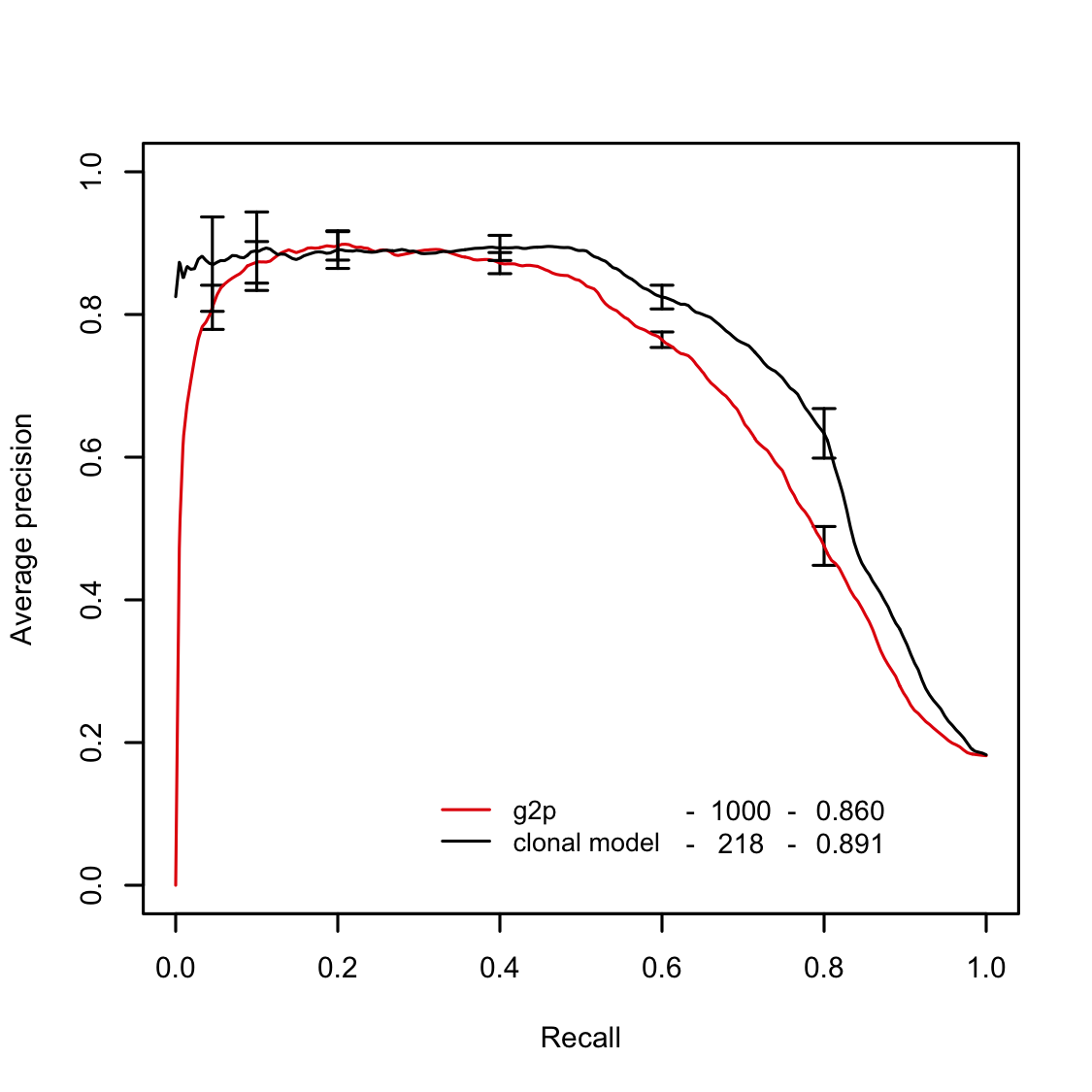

Supplement: Figure S14 — Separation of amino acid indices into 12 clusters – the separation that showed the largest silhouette value. (TIFF) [file pcbi.1002977.s014.tif]
