# Supplementary material for: Analysis of Physicochemical and Structural Properties Determining HIV-1 Coreceptor Usage
Source: PLoS Comput Biol. 2013 Mar 21;9(3):e1002977. doi: 10.1371/journal.pcbi.1002977 (PMC3605109; doi:10.1371/journal.pcbi.1002977)
Supplement: Table S1 — Performance of the clinical model and models derived from the clinical dataset by removing sequences with ambiguities (HOMER-filter), removing sequences without ambiguities (HOMER-ambi) and replacing ambiguities with gaps (HOMER-gap). (PDF) [file pcbi.1002977.s015.pdf]

dataset	sequences (X4 virus)	features	AUC	sensitivity
HOMER	954 (167)	59	0.774	0.463
HOMER-filter	412 (39)	95	0.657	0.313
HOMER-ambi	542 (128)	22	0.794	0.288
HOMER-gap	954 (167)	106	0.774	0.303
