# Supplementary material for: Analysis of Physicochemical and Structural Properties Determining HIV-1 Coreceptor Usage
Source: PLoS Comput Biol. 2013 Mar 21;9(3):e1002977. doi: 10.1371/journal.pcbi.1002977 (PMC3605109; doi:10.1371/journal.pcbi.1002977)
Supplement: Text S1 — Selection of model parameters. (PDF) [file pcbi.1002977.s021.pdf]

Selection of model parameters: sphere radius and Gaussian smoothing function

The radius of the spheres was chosen such as to provide a balance between too small proximities that do not incorporate structural information and too large proximities that reduce spatial resolution. Black histograms in Figure S1 represent the distribution of the number of residues included in proximities of a radius indicated on the corresponding plot on the left. Spheres of radius 6 include five residues on average with a 4.56 variance among spheres that might be insufficient for representing a structural binding site. On the other hand, spheres of size 12 contain 16 residues on average, which corresponds to almost half of the loop. Sphere radius 8 was chosen for further testing. Spheres of this radius contain 8.4 residues on average with a relatively small variability among spheres. We additionally tested other radii (3Å, 7Å, 10Å, 15Å) to assess the performance of the chosen parameter value (Figure S1).

Each V3 sequence position was mapped to a sphere if the corresponding representative atom was located within the given sphere. Within each sphere the vectors of amino acid indices of the mapped residues were normalized using Gaussian smoothing. The vector of amino acid indices representing a mapped residue was multiplied by the value of a normalized Gaussian function applied to the distance between the representative atom of the residue and the sphere center. We inspected the cumulative contribution of each V3 residue to the descriptor as a function of the variance of the Gaussian function (Figure S1). Red histograms in Figure S1 illustrate the sum of Gaussian normalizing factor per each residue. This sum is comparable for each residue therefore no individual residue is weighted markedly higher than others in the structural descriptor. The narrowest distribution was obtained for the variation parameter equal to the radius ($R = 8$, $var = 8$). We chose the variance equal to the sphere radius as resulting in the most uniform contribution of each residue to the descriptor and therefore not giving priority to any of the loop residues. We tested an additional set of variances (ratio 0.5 and 0.75 of the sphere radius) to assess the impact of this parameter on prediction performance.
