# Supplementary material for: Analysis of Physicochemical and Structural Properties Determining HIV-1 Coreceptor Usage
Source: PLoS Comput Biol. 2013 Mar 21;9(3):e1002977. doi: 10.1371/journal.pcbi.1002977 (PMC3605109; doi:10.1371/journal.pcbi.1002977)
Supplement: Text S2 — Overlap of features selected by different feature selection methods. (PDF) [file pcbi.1002977.s022.pdf]

Overlap of features selected by different feature selection methods

Feature sets selected using the three selection methods RF, SVM and Lasso show a limited overlap of approximately 1% between SVM and RF, 3.5% between SVM and Lasso and approximately 9% between RF and Lasso. Features in the initial feature set showed low general correlation (median 0.03, see next section). A plausible reason for the low overlap of features selected by different methods is correlation of features describing similar amino acid properties in an overlapping region on the structure. Each alignment position falls into 16.6 spheres on average, between 5 at the start and end of the V3 loop and 25 spheres in the middle part of the loop where its two sides are in close vicinity. Notably, most of the selected features that are important for tropism are located in the central part of the loop. Due to the spatial overlap of the spheres certain features pertaining to overlapping spheres convey similar information. Different feature selection methods might therefore select different features out of groups of such highly correlated ones.

We inspected correlation and positions in the structure of the highly correlated features selected by pairs of methods: SVM-RF, SVM-Lasso, RF-Lasso. For each feature selected by one method we searched for three most highly correlated features among those selected by the other method from the given pair of methods and tested how closely in the structure they are located. The highly correlated features are mostly located in overlapping spheres (Figure S3) and are significantly more closely located than arbitrary features ($p < 0.01$, Wilcoxon test corrected for multiple testing). Negatively correlated features show similar colocalization (Figure S3) which explains the low overlap of feature set, however might provide different or complementary biological insights

This suggests that the low overlap of features selected by different feature selection methods might be due to the local correlation of features. Despite the low overlap of sets of features selected using different methods, these sets provide similar information regarding the physicochemical properties of the V3 loop determining coreceptor usage.
