# Supplementary material for: Analysis of Physicochemical and Structural Properties Determining HIV-1 Coreceptor Usage
Source: PLoS Comput Biol. 2013 Mar 21;9(3):e1002977. doi: 10.1371/journal.pcbi.1002977 (PMC3605109; doi:10.1371/journal.pcbi.1002977)
Supplement: Text S3 — Feature correlation. (PDF) [file pcbi.1002977.s023.pdf]

Feature correlation

Correlation of features is known to potentially reduce the predictive accuracy of the underlying model[1]. There are several approaches to reducing of feature correlation, e.g. Tolosi et al[2]. However, in general, the correlation of features in the initial feature set in our study is low (Figure S4) and the models constructed on features clustered with the methods of Tolosi et al[2] showed no improvement in accuracy over the models based on original feature set. Therefore, for the purpose of higher interpretability of our models, we chose the more parsimonious approach not involving feature clustering for our study.

---

[1] HastieT, Tibishirani R, Friedman J. *The elements of statistical learning*. Chapter 3, Springer 2009

[2] Tolosi L, Lengauer T (2011) *Classification with correlated features: unreliability of feature ranking and solutions*. Bioinformatics Jul 15;27(14):1986-94
