# Supplementary material for: Analysis of Physicochemical and Structural Properties Determining HIV-1 Coreceptor Usage
Source: PLoS Comput Biol. 2013 Mar 21;9(3):e1002977. doi: 10.1371/journal.pcbi.1002977 (PMC3605109; doi:10.1371/journal.pcbi.1002977)
Supplement: Text S4 — Combining structure and sequence descriptors. (PDF) [file pcbi.1002977.s024.pdf]

Combining structure and sequence descriptors.

In order to test how much performance can be gained through incorporation of a different V3 loop structure, we repeated the same model construction and feature selection procedure using a more recently reported V3 loop structure (Protein Data Bank (PDB) code 2QAD[1]). The descriptor vectors based on the two structures had a mean correlation of 0.71 ($p < 0.01$) for the V3 loops in the clonal dataset, which reflects high similarity of the two descriptors. The models based on the 2QAD structure showed a similar performance to those based on the initially used 2B4C structure. The 2QAD-based SVM(1)_Lasso model showed an AUC of 0.872 and sensitivity of 0.639. The selected features of models based on the two structures had a significant ($p < 0.01$) overlap of 10% for the features selected through SVM, 10-17% for Lasso- and 20-23% for the RF-selected features, which additionally demonstrates the similarity of models based on these two structures.

Next, we tested the performance of combinations of structure- and sequence-based descriptors. A combination of descriptors was obtained by concatenating the vectors of the respective descriptors. As already suggested by the high correlation of the two structural descriptors, their combination did not yield an improvement in prediction performance. The combination of the SVM(1)_Lasso models based on two different structures achieved an AUC 0.857 and sensitivity 0.625. Combining the sequence-based binary descriptor with the 2B4C-based structural descriptor or with the combined structural descriptors in both cases resulted in AUC 0.875 and sensitivity 0.630. We concluded that combining the clonal model with additional sequence- or structure-based descriptors does not result in improved performance.

---

[1] Huang CC, Lam SN, Acharya P, Tang M, Xiang SH, et al. (2007) *Structures of the CCR5 N terminus and of a tyrosine-sulfated antibody with HIV-1 gp120 and CD4.* Science 317: 1930-1934
