# Supplementary material for: Analysis of Physicochemical and Structural Properties Determining HIV-1 Coreceptor Usage
Source: PLoS Comput Biol. 2013 Mar 21;9(3):e1002977. doi: 10.1371/journal.pcbi.1002977 (PMC3605109; doi:10.1371/journal.pcbi.1002977)
Supplement: Text S5 — Validation of the clinical model on external dataset. (PDF) [file pcbi.1002977.s025.pdf]

Validation of the clinical model on an external dataset

Given the uncertainties pertaining to the patient-derived bulk sequence data, we compared the performances of the proposed structure-based method and sequence-based g2p model on an additional dataset external to this study (hivgrade dataset). This dataset contains 760 patient-derived clinical samples sequenced in bulk and phenotyped using standard Trofile (620 sequences) and enhanced Trofile (140 sequences) assays. 34% of the sequences showed X4 phenotype and were equally distributed between sequences phenotyped by the two types of assay. We predicted the phenotypes of the sets of sequences phenotyped by both assays using the structure-based clinical model and g2p model trained on the clinical dataset. The results of this comparison are shown in Figure S6.

According to the provider, the enhanced Trofile assay is more sensitive to X4 variants than the standard Trofile assay. In the subset phenotyped by the enhanced Trofile assay, the structure-based model is clearly better performing when trained on clinical data (AUC 0.748, sensitivity 0.345) than when trained on clonal data (AUC 0.634, sensitivity 0.273). In addition, the structure-based clinical model performs better than the sequence-based g2p models trained on clonal data (AUC 0.711, sensitivity 0.291) or trained on clinical data (AUC 0.665, sensitivity 0.291). These differences in performance between the clinical and clonal models are not observed on the subset of sequences phenotyped with the less sensitive standard Trofile assay. Nevertheless, in this subset both the clinical and clonal structure-based models outperform the corresponding sequence-based models with 0.769 AUC, sensitivity 0.527 vs. 0.750 AUC, 0.371 sensitivity of the models train on clinical data and 0.801 AUC, sensitivity 0.507 vs. 0.776 AUC, 0.527 sensitivity of the models trained on clonal data.
