# Supplementary material for: Analysis of Physicochemical and Structural Properties Determining HIV-1 Coreceptor Usage
Source: PLoS Comput Biol. 2013 Mar 21;9(3):e1002977. doi: 10.1371/journal.pcbi.1002977 (PMC3605109; doi:10.1371/journal.pcbi.1002977)
Supplement: Text S6 — Effect of ambiguities on prediction accuracy. (PDF) [file pcbi.1002977.s026.pdf]

Effect of ambiguities on the prediction accuracy

As previously shown[1], genotypic methods for tropism prediction show lower accuracy on clinically derived data than on clonal data. This might be due to noise introduced by ambiguous positions and to the presence of minorities undetectable by bulk genotyping of viral populations. In order to test whether our strategy of handling sequence ambiguities impacts prediction accuracy we tested two sequence sets derived from the original dataset and not containing ambiguities. The *HOMER-filter* dataset was obtained from the HOMER dataset after removing all sequences containing ambiguous positions. It contains 412 sequences with 39 X4 virus sequences. The *HOMER-ambi* dataset is the complement of the HOMER-filter dataset and groups all sequences that contain ambiguous positions. The *HOMER-gap* dataset was derived from the HOMER dataset by replacing all ambiguous positions with gaps. The same steps involving construction of structural descriptors, feature selection using Lasso and evaluation using cross validation were performed on the two datasets not containing ambiguous positions. Lower prediction performance was observed on all datasets (Table S1), suggesting that combined information from both types of positions is important for tropism prediction and that the presence of undetectable minorities and lack of information on the exact composition of virus variants in a population might be the reason for the low prediction accuracy of models applied to clinically derived data.

---

[1] Sing T, Low AJ, Beerenwinkel N, Sander O, Cheung PK, et al. (2007) *Predicting HIV coreceptor usage on the basis of genetic and clinical covariates*. Antivir Ther 12: 1097-1106
